# Changes in the critical nutrient content of packaged foods and beverages after the full implementation of the Chilean Food Labelling and Advertising Law: a repeated cross-sectional study

**DOI:** 10.1186/s12916-025-03878-6

**Published:** 2025-01-27

**Authors:** Natalia Rebolledo, Pedro Ferrer-Rosende, Marcela Reyes, Lindsey Smith Taillie, Camila Corvalán

**Affiliations:** 1 Center of Research in Food Environment and Prevention of Obesity and Non-Communicable Diseases (CIAPEC), Institute of Nutrition and Food Technology (INTA), University of Chile, Santiago, Chile; 2 Biostatistics Unit, Faculty of Medicine, Universität Autònoma de Barcelona. Cardanol del Vallès, Barcelona, Spain; 3 Carolina Population Center, Department of Nutrition, Gillings School of Global Public Health, University of North Carolina at Chapel Hill, Chapel Hill, NC USA

**Keywords:** Food reformulation, Chile Food Labelling Law, Food environment regulation, Critical nutrients

## Abstract

**Background:**

Chile’s Food Labelling Law was implemented in three phases with increasingly stricter limits. After initial implementation, sugars and sodium decreased in packaged foods, with no significant changes for saturated fats. It is unclear whether full implementation is linked with further reformulation or if producers reversed changes due to consumers’ preferences. This study examines changes in the proportion of “high in” products and the nutrient content of packaged foods during the Law’s three phases.

**Methods:**

This repeated cross-sectional study included the best-selling packaged foods and beverages during 2015–2020. We analyzed the proportion of products classified as “high in” critical nutrients using the final phase cutoffs and examined changes in the content of calories, sugars, sodium, and saturated fats in the three phases. To assess the changes in proportions, we used Firth’s bias-reduced logistic regression models and the Cochran-Armitage test for trends. Quantile regression was used to evaluate changes in nutrient content.

**Results:**

The proportion of “high in” products decreased from 70.8 to 52.5% after the final phase (*p* < 0.001). The proportion of “high in” sugars products decreased across all sweet food and beverage groups (*p* < 0.001), except for candies (− 4.5 percentage points (pp), *p* = 0.09). The largest reductions occurred in sweet spreads and breakfast cereals (− 44.3 and − 40.4 pp, respectively, *p* < 0.001). For the proportion of “high in” sodium, reductions occurred in all savory food groups (*p* < 0.001), except cheeses and ready-to-eat meals (*p* < 0.24), with the largest decreases in savory baked products and non-sausage meat products (− 40.4 and − 38.9 pp, respectively, *p* < 0.001). Reductions in “high in” saturated fats and energy were less consistent, with the largest decreases in nuts and snacks and savory spreads (− 22.2 and − 20.0 pp, respectively, *p* < 0.001) and savory baked products and breakfast cereals (− 32.8 and − 25.7 pp, respectively, *p* < 0.001), respectively. After full implementation, most sweet categories showed left shifts in sugars distribution, except for candies. Similarly, most savory categories showed left shifts for sodium, except savory spreads and ready-to-eat meals. Changes increased as regulation limits tightened (*p* for trend < 0.001).

**Conclusions:**

After fully implementing Chile’s law, the proportion of “high in” products and the content of critical nutrients decreased in all food and beverage categories. The largest changes occurred for sodium in savory foods and sugars in sweet foods/beverages. Stricter regulatory limits were associated with decreases in critical nutrient content over time.

**Supplementary Information:**

The online version contains supplementary material available at 10.1186/s12916-025-03878-6.

## Background

In June 2016, Chile was the first country to implement a unique regulation that combines three actions to promote healthier food environments [1]. The Food Labelling and Advertising Law includes the use of black octagonal warning labels on the front-of-package of unhealthy foods to inform consumers that certain products have a high content of calories or added nutrients associated with an increased risk of nutrition-related chronic diseases (NCDs) (i.e., saturated fats, total sugars, and sodium). Then, there are comprehensive restrictions to decrease the exposure to the marketing of unhealthy foods for children under 14 years. Finally, unhealthy food products and beverages cannot be sold, promoted, or distributed for free (i.e., in school feeding programs) in school environments [1]. The regulation was implemented in three phases, defining limits for the regulated nutrients that became increasingly stricter over four years.

Previous studies have shown that after the initial implementation of the regulation, there were important changes in the food environment [2, 3]. These changes were associated with a decrease in purchases of “high in” foods and beverages, resulting in reductions in purchases of calories, sugars, sodium, and saturated fats due to both product reformulation and changes in purchasing behavior [4, 5]. In the food supply, after the initial implementation, we observed important reductions in the content of total sugars and sodium in some food groups, such as beverages, milk and milk-based products, breakfast cereals, sweets spreads, and savory spreads, among others [6]. Given that the regulation limits became stricter between the initial and final phases, it is unclear whether the implementation of the following phases of the regulation was associated with further changes in the food supply. For example, the initial implementation limits for solids per 100 g were 350 kcal, 22.5 g of sugars, 6 g of saturated fats, and 800 mg of sodium. After full implementation, these limits were reduced to 275 kcal, 10 g of sugars, 4 g of saturated fats, and 400 mg of sodium. The stricter limits could lead either to progressive reformulation or a limitation of the reformulation effort.

Assessing the overall changes after the regulation is relevant because it provides insight into whether stricter limits are associated with further reformulation. Additionally, there is a concern that some changes in the nutrient composition of food products may not be sustained if consumers do not adapt their food preferences at a similar speed. Therefore, in the current study, we aimed to examine changes in the proportion of regulated products and changes in energy, total sugars, saturated fats, and sodium in packaged foods and beverages before (2015–2016) and after each of the three regulatory phases (2017, 2019, 2020) of the Chilean Food Labelling and Advertising Law. While the observational nature of this study cannot establish causality, it provides valuable insights into the changes in the nutritional composition of food products during the law’s three phases.

## Methods

### Summary of study design

**Fig. 1:**
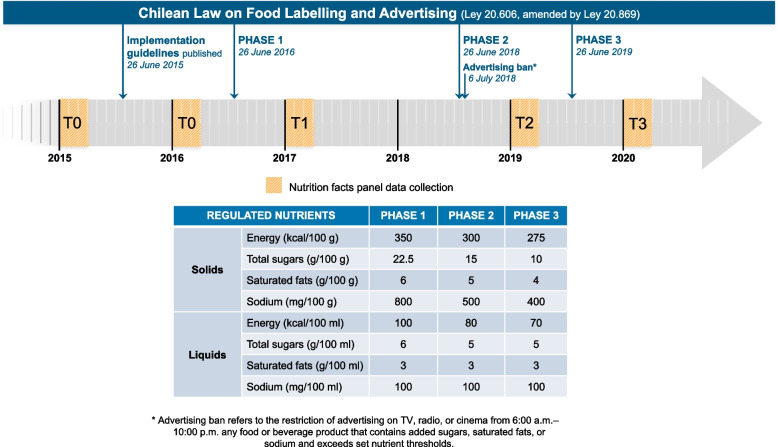
Timeline of the implementation of the Chilean Law of Food Labelling and Advertising and data collection. T0, preimplementation period; T1, postimplementation of the 1st phase of the law; T2, postimplementation of the 2nd phase of the law; T3, postimplementation of the 3rd phase of the law

This is a prospective repeated cross-sectional study. Nutrition facts panel data were collected annually from 2015 to 2020 in supermarkets in Santiago, Chile. We defined four periods corresponding to the phases of the Chilean law: pre-law (T0: 2015–2016), initial phase (T1: 2017), second phase (T2: 2019), and final phase (T3: 2020). Each regulatory phase (T1, T2, and T3) was compared with the pre-law period (T0). A timeline of the data collection and the implementation of the phases of the law is available in Fig. 1.

The analytic sample included the best-selling packaged foods and beverages with sales ≥ 1% within their food groups, according to Euromonitor International Database food categories available in 2022 [7]. These products accounted for over 95% of the total market share, except for chocolate confectionery and sweet biscuits (~ 80%, Additional File 1: Table S1). Sensitivity analyses, including all products collected, irrespective of their market share, are also presented in Additional File 1. Products were categorized as “high in” if they contained added sugars, saturated fats, or sodium and surpassed the nutrient limits set by the final phase of the law (Fig. 1). Changes in the proportion of “high in” products were analyzed using Firth’s bias-reduced logistic regression models, while changes in the content of calories, total sugars, sodium, and saturated fats were assessed using quantile regression.

This study expands on our previous work assessing the reformulation of foods and beverages during the initial implementation of the law (T0 vs T1) [6]. While the earlier study analyzed both longitudinal and cross-sectional samples, showing consistent results, the current analysis is restricted to repeated cross-sectional samples. This adjustment was necessary given the decrease in the sample size for foods and beverages collected longitudinally through T0, T1, T2, and T3. Additionally, while the earlier study applied nutrient and calorie thresholds specific to the first phase of the law (available in Fig. 1), the current study uniformly applies the final phase’s nutrient thresholds across all phases. This approach ensures a consistent basis for comparison over time (i.e., using the same criteria to define “high in” foods). However, it does not reflect the actual thresholds used at that time according to the staggered implementation strategy. Also, during the 2020 data collection wave, some supermarkets were replaced (details provided below). For statistical analysis, we used Firth’s bias-reduced logistic regression to assess the differences in the proportion of “high in” products instead of the chi-squared test. Apart from these distinctions, most methodological elements remained consistent between the two studies, including the criteria for data collection, the definition of best-selling products, and exclusion parameters. These methodological decisions are relevant when interpreting current results and comparing both studies.

### The Chilean Food Labelling and Advertising Law

Chile’s law requires packaged foods and beverages with added sugars, sodium, or saturated fats and exceeding defined thresholds for these nutrients or caloric content to carry front-of-package warning labels with the words “high in” sugars, sodium, saturated fats, and/or calories. The labels are black and white stop signs, and each packaged food and beverage can carry up to four labels (Additional File 1: Fig. S1). “High in” products are subject to marketing restrictions for children and banned from sale or promotion in schools and nurseries. The law was implemented in three phases (2016, 2018, and 2019), with progressively restrictive nutrient thresholds for solids and liquids (Fig. 1). It is important to note that small and very small food producers were granted a three-year delay in implementing the law [1]. Therefore, those products began to be labelled in June 2019 using the first-phase thresholds. Micro-enterprises are exempt from the regulation until June 2026 [8].

### The Chilean Nutrition Facts Panel (NFP) database

The NFP database contains nutrition information for packaged products in the Chilean food supply. These data were obtained from photographs of products that a team of Chilean nutrition research assistants collected in stores located in Santiago between 2015 and 2020. The data collection methods have been previously described in the literature [9]. Briefly, data were collected in annual waves from January to February each year. Photographs were collected from 6 to 8 major supermarkets due to an agreement with the Chilean National Association of Supermarkets (ASACH). The selected supermarkets represented one of the six major national supermarket chains, known for having a greater variety of food products. Three candy distributors were also included to increase the variety of candies and sweet confectioneries. Given the protests in Chile in the final quarter of 2019, we slightly modified the 2020 data collection because some supermarkets remained closed during the data collection period in January and February (before the COVID-19 pandemic). We replaced these supermarkets with others from the same chain that were geographically close to the previously visited locations.

During the fieldwork, research assistants took four mandatory photos of each packaged product available in the supermarket: the front of the package, the nutrition facts panel, the list of ingredients, and the warning labels (or a note indicating “no warning label”). After data collection, trained dietitians reviewed the photos and entered general identifying information for each product on a software that our team developed for this purpose. Information coded included: general information such as barcode, brand, flavor or other important identifier details, manufacturer, among others, presence of labels, ingredients list, amount of energy and nutrients (i.e., protein, carbohydrates, total sugars, total fats, fat subtypes if available, sodium, micronutrients, nonnutritive sweeteners) per 100 g (g) or 100 ml (mL), and reconstitution instructions when available. Quality control checks were conducted throughout the period by a supervisor and pictures were rechecked for accuracy in the data entry.

### Food and beverage groups

Each food and beverage available in the NFP data was categorized into one of 16 mutually exclusive food groups based on adapted classifications used in previous research [6]. These groups were created to visualize the potential changes in regulated nutrients. Therefore, we separated solids from liquids due to different regulation thresholds. The groups for this research were as follows: beverages (sugar-sweetened, nonnutritive-sweetened, and unsweetened); milks and milk-based drinks; yogurts; breakfast cereals (ready-to-eat and to be prepared); sweet baked products; desserts, ice creams, and processed fruits; candies and sweet confectionery; sweet spreads; savory baked products; nuts and snacks; savory spreads, seasonings, and dressings; cheeses; ready-to-eat meals; sausages; non-sausage meat products; and soups (powdered and ready-to-eat).

### Data processing and definition of the analytical sample

**Fig. 2:**
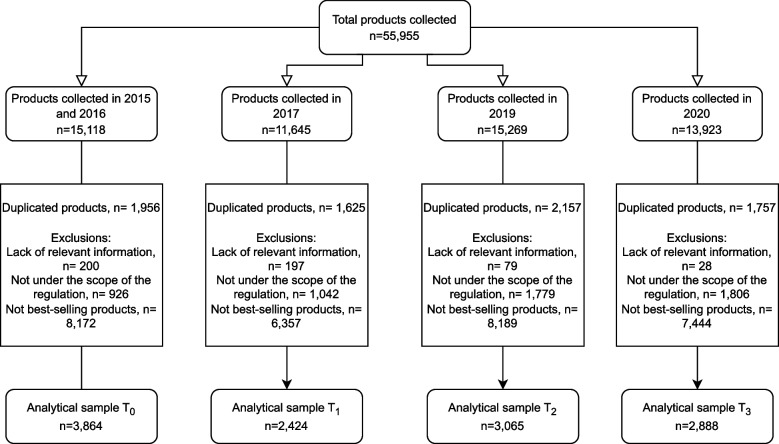
Flow chart describing products excluded from the analytical sample. T0, preimplementation period; T1, postimplementation of the 1st phase of the law; T2, postimplementation of the 2nd phase of the law; T3, postimplementation of the 3rd phase of the law. Products were collected based on their availability in supermarkets each year

Figure 2 shows the number of products included and excluded from the analytical sample. A total of 55,955 products were photographed across five data collection waves conducted between 2015 and 2020. All data collected in 2015 and 2016 (pre-policy implementation, T0) were pooled to build a larger baseline dataset. We retained the most recent product in case of duplicates (i.e., only items collected in 2016 were included). Data collected in 2017, 2019, and 2020 represented the initial phase (T1), second phase (T2), and final phase (T3) of the law, respectively. The number of products photographed in each period ranged from 11,645 in 2017 (T1) to 15,269 in 2019 (T2).

Within each of the cross-sectional samples, we collected different package sizes for the same product within a year (i.e., a beverage could have been collected in 2 or 3 L size). However, given that these products had the same nutritional information, ingredients list, and Euromonitor category, we considered them duplicates. We excluded the duplicates from the final dataset (12.9% for T0 or 2015–2016, 14.0% for T1 or 2017, 14.1% for T2 or 2019, and 12.6% for T3 or 2020). We also excluded products that lacked relevant information (i.e., missing ingredients list, any information on the amount of energy and nutrients, reconstitution instructions when needed; 1.3% for T0, 1.7% for T1, 0.5% for T2 and 0.2% for T3). For saturated fats, missing values were replaced by 0 when the amount of total fats was below 3 g per portion size because the Chilean regulations require specific fats to be declared only if the content of total fats is equal or above 3 g per serving [10]. Additionally, we excluded products outside the scope of the regulation (i.e., unprocessed and minimally processed foods, culinary ingredients without an increase in the natural content of regulated nutrients as part of their processing, infant formulas, and baby foods without added sugars; 6.1% for T0, 8.9% for T1, 11.7% for T2, and 13.0% for T3). Lastly, we also excluded not best-selling products (i.e., < 1% market share within each of the 32 main food groups from the Euromonitor database available in 2022) [7]; 54.1% for T0, 54.6% for T1, 53.6% for T2, and 53.5% for T3). For each year, market share within a specific food group was computed as Euromonitor sales of < product or brand family of products > during < year > × 100 ÷ addition of sales of < Euromonitor food group > . Products meeting the ≥ 1% of the market share of their food group were manually selected from the list of Euromonitor products (or brand family of products if the product was not directly available). The final analytic sample included 3864 products for T0, 2424 products for T1, 3065 products for T2, and 2888 products for T3.

### Food and beverage products with high content of critical nutrients

Packaged products under the scope of the regulation (i.e., those containing added sugars, saturated fats, or sodium in the list of ingredients) [11] were defined as having a high content of critical nutrients (i.e., “high in” products) if their energy and nutrient contents exceeded the cutoffs of the final phase of the law (Fig. 1). For products requiring reconstitution (e.g., powdered milk, powdered soups, concentrate juices, among others), we used the package instructions to calculate the energy content and regulated nutrients in the product as consumed.

We applied the final phase limits to the foods and beverages collected at T0, T1, T2, and T3, regardless of the phase-specific limits that were applicable at the time. This approach allowed us to assess how the percentage of products meeting these thresholds and their critical nutrient content in the food supply changed throughout the implementation of the regulation, using the limits of the final phase as a benchmark for defining food products with high critical nutrient content. The cutoffs for solids or liquids were used depending on the unit of measure displayed on the label (g for solids or mL for liquids). For reconstituted products, we used the limits for liquids.

### Data analyses

The analysis plan was preregistered on January 20, 2022, and is available at https://osf.io/n2y87.

### Outcomes

The primary outcomes were changes in the proportion of products exceeding the final phase limits for energy, total sugars, saturated fats, sodium, or any “high in” (i.e., products high in energy or at least one nutrient of concern) and changes in the quartiles of energy, total sugars, saturated fats, and sodium (amount per 100 g or 100 mL). These changes were assessed across all products, separately for solids and liquids, and within specific food and beverage groups. Comparisons were made between the pre-law period (T0) and each of the following phases of the law (T1, T2, and T3).

### Statistical analyses

All statistical analyses were conducted on R v4.1.3 (RCoreTeam, 2022, Vienna, Austria). In the repeated cross-sectional analysis, we assessed changes in the proportion of “high in” products by contrasting the estimated marginal means from Firth’s bias-reduced logistic regression models. The Cochran-Armitage test was used to evaluate whether these changes followed a trend over time. Firth’s method was chosen because it adjusts the likelihood function to address biases that arise when dealing with zero or near-zero values, which can lead to unreliable or infinite estimates in standard logistic regression or other methods. By modifying the likelihood, Firth’s correction provides more stable and accurate estimates [12]. However, the contrasts should be interpreted with caution, as they represent pairwise post-hoc comparisons. Finally, we employed quantile regressions with the implementation period as the independent variable to estimate changes in the 25th, 50th, and 75th percentiles of energy or the nutrient of concern by food or beverage group. Density plots were generated to illustrate the distribution of energy and the relevant nutrients for specific food or beverage groups for each implementation phase, allowing visual comparison of the changes that each regulation phase.

### Sensitivity analyses

The primary analyses focused on best-selling products to capture reformulation in food and beverage products that are more relevant from a consumption perspective. We consider that this is a more relevant analysis from a public health perspective. Nonetheless, we also included full supply analyses, which are available in Additional File 1 (Fig. S2, Tables S2-S4).

## Results

### Changes in the proportion of foods and beverages with high content of critical nutrients

Table 1 shows the changes in the proportion of “high in” products overall and for solids and liquids, and Table 2 shows the changes in the proportion of “high in” products by food and beverage groups, categorized according to the nutrient limits of the final phase of the law (i.e., full implementation).

**Table 1:** Changes in the proportion of “high in” energy and nutrients of concern (or any "high in") before (T0) and after each phase of Chile’s Law (T1, T2, T3) for solid and liquid products, repeated cross-sectional analysis of best-selling items

	**2016 (T**_**0**_**)**	**2017 (T**_**1**_**)**	**2019 (T**_**2**_**)**	**2020 (T**_**3**_**)**	**Difference (T0 vs T3)**		*p*-trend**
					**Absolute**	*p*-value*	
**Overall**	*N* = 3864	*N* = 2424	*N* = 3065	*N* = 2888			
Any “high in”^abcde^	2735 (70.8%)	1536 (63.4%)	1671 (54.5%)	1517 (52.5%)	− 18.3	< 0.001	< 0.001
High in energy^abcde^	1365 (35.3%)	922 (38.0%)	976 (31.8%)	931 (32.2%)	− 3.1	0.008	< 0.001
High in sugars^bcde^	1313 (34.0%)	804 (33.2%)	780 (25.4%)	679 (23.5%)	− 10.5	< 0.001	< 0.001
High in saturated fats^bcde^	1071 (27.7%)	658 (27.1%)	721 (23.5%)	664 (23.0%)	− 4.7	< 0.001	< 0.001
High in sodium^abcef^	1300 (33.6%)	644 (26.6%)	800 (26.1%)	665 (23.0%)	− 10.6	< 0.001	< 0.001
**Solids**	*N* = 2547	*N* = 1694	*N* = 2036	*N* = 1875			
Any “high in”^abcdef^	2051 (80.5%)	1290 (76.2%)	1434 (70.4%)	1253 (66.8%)	− 13.7	< 0.001	< 0.001
High in energy^ade^	1175 (46.1%)	875 (51.7%)	921 (45.2%)	873 (46.6%)	0.5	0.778	0.240
High in sugars^acde^	873 (34.3%)	668 (39.4%)	660 (32.4%)	560 (29.9%)	− 4.4	0.002	< 0.001
High in saturated fats^bcde^	971 (38.1%)	625 (36.9%)	682 (33.5%)	621 (33.1%)	− 5.0	< 0.001	< 0.001
High in sodium^abcef^	1066 (41.9%)	542 (32.0%)	697 (34.2%)	537 (28.6%)	− 13.3	< 0.001	< 0.001
**Liquids**	*N* = 1317	*N* = 730	*N* = 1029	*N* = 1013			
Any “high in”^abcde^	684 (51.9%)	246 (33.7%)	237 (23.0%)	264 (26.1%)	− 25.8	< 0.001	< 0.001
High in energy^abc^	190 (14.4%)	47 (6.4%)	55 (5.3%)	58 (5.7%)	− 8.7	< 0.001	< 0.001
High in sugars^abcde^	440 (33.4%)	136 (18.6%)	120 (11.7%)	119 (11.7%)	− 21.7	< 0.001	< 0.001
High in saturated fats^abc^	100 (7.6%)	33 (4.5%)	39 (3.8%)	43 (4.2%)	− 3.4	0.001	< 0.001
High in sodium^abcd^	234 (17.8%)	102 (14.0%)	103 (10.0%)	128 (12.6%)	− 5.2	< 0.001	< 0.001
**Only beverages and milks and milk-based drinks**	*N* = 933	*N* = 581	*N* = 826	*N* = 781			
Any “high in”^abcde^	310 (33.2%)	116 (20.0%)	77 (9.3%)	81 (10.4%)	− 22.8	< 0.001	< 0.001
High in energy^bcde^	24 (2.6%)	11 (1.9%)	3 (0.4%)	3 (0.4%)	− 2.2	0.002	0.003
High in sugars^abcde^	307 (32.9%)	116 (20.0%)	76 (9.2%)	81 (10.4%)	− 22.5	< 0.001	< 0.001
High in saturated fats	0 (0.0%)	4 (0.7%)	0 (0.0%)	0 (0.0%)	0.0	0.929	0.226
High in sodium	4 (0.4%)	3 (0.5%)	1 (0.1%)	0 (0.0%)	− 0.4	0.175	0.057
**Other liquids**	*N* = 384	*N* = 149	*N* = 203	*N* = 232			
Any “high in”^abcde^	374 (97.4%)	130 (87.2%)	160 (78.8%)	183 (78.9%)	− 18.5	< 0.001	< 0.001
High in energy^abc^	166 (43.2%)	36 (24.2%)	52 (25.6%)	55 (23.7%)	− 19.5	< 0.001	< 0.001
High in sugars^abc^	133 (34.6%)	20 (13.4%)	44 (21.7%)	38 (16.4%)	− 18.2	< 0.001	< 0.001
High in saturated fats^c^	100 (26.0%)	29 (19.5%)	39 (19.2%)	43 (18.5%)	− 7.5	0.035	0.002
High in sodium^bde^	230 (59.9%)	99 (66.4%)	102 (50.2%)	128 (55.2%)	− 4.7	0.250	0.001

Values represent the sample size and the proportion of regulated products

Cutoffs correspond to the limits on the amount of energy or nutrient of concern for the full implementation of the law (i.e., for solids, per 100 g: 275 kcal of energy, 10 g of sugars, 4 g of saturated fats, 400 mg of sodium; for liquids, per 100 mL: 70 kcal of energy, 5 g of sugars, 3 g of saturated fats, 100 mg of sodium)

T0: preimplementation period, January to February 2015 + January to February 2016 (*n* = 3864); T1: postimplementation of the 1st phase of the law, January to February 2017 (*n* = 2424); T2: postimplementation of the 2nd phase of the law, January to February 2019 (*n* = 3065); T3: postimplementation of the 3rd phase of the law, January to February 2020 (*n* = 2888)

^a^*p*-value < 0.05. comparisons between T0 and T1 were made by contrasting estimated marginal means (EMMs) from Firth’s bias-reduced logistic regression

^b^*p*-value < 0.05. comparisons between T0 and T2 were made by contrasting estimated marginal means (EMMs) from Firth’s bias-reduced logistic regression

^c^*p*-value < 0.05. comparisons between T0 and T3 were made by contrasting estimated marginal means (EMMs) from Firth’s bias-reduced logistic regression

^d^*p*-value < 0.05. comparisons between T1 and T2 were made by contrasting estimated marginal means (EMMs) from Firth’s bias-reduced logistic regression

^e^*p*-value < 0.05. comparisons between T1 and T3 were made by contrasting estimated marginal means (EMMs) from Firth’s bias-reduced logistic regression

^f^*p*-value < 0.05. comparisons between T2 and T3 were made by contrasting estimated marginal means (EMMs) from Firth’s bias-reduced logistic regression

^*^p-value < 0.05. comparisons between T0 and T3 were made by contrasting estimated marginal means (EMMs) from Firth’s bias-reduced logistic regression

^**^p-value for Cochrane Armitage Test for trend

**Table 2:** Changes in the proportion of “high in” energy and nutrients of concern (or any "high in") before (T0) and after each phase of Chile’s Law (T1, T2, T3) by food and beverage group, repeated cross-sectional analysis of best-selling items

	**2016 (T**_**0**_**)**	**2017 (T**_**1**_**)**	**2019 (T**_**2**_**)**	**2020 (T**_**3**_**)**	**Difference (T0 vs T3)**		*p*-trend**
					**Absolute**	*p*-value*	
**Beverages**	*N* = 756	*N* = 545	*N* = 725	*N* = 630			
Any “high in”^abcde^	221 (29.2%)	112 (20.6%)	68 (9.4%)	71 (11.3%)	− 17.9	< 0.001	< 0.001
High in energy^ade^	8 (1.1%)	14 (2.6%)	4 (0.6%)	0 (0.0%)	− 1.1	0.068	0.002
High in sugars^abcde^	219 (29.0%)	112 (20.6%)	67 (9.2%)	71 (11.3%)	− 17.7	< 0.001	< 0.001
High in saturated fats	1 (0.1%)	5 (0.9%)	1 (0.1%)	0 (0.0%)	− 0.1	0.574	0.170
High in sodium	2 (0.3%)	3 (0.6%)	0 (0.0%)	0 (0.0%)	− 0.3	0.356	0.055
**Milks and milk-based drinks**	*N* = 197	*N* = 98	*N* = 169	*N* = 155			
Any “high in”^abc^	93 (47.2%)	9 (9.2%)	11 (6.5%)	11 (7.1%)	− 40.1	< 0.001	< 0.001
High in energy^abc^	18 (9.1%)	0 (0.0%)	1 (0.6%)	3 (1.9%)	− 7.2	0.012	< 0.001
High in sugars^abc^	91 (46.2%)	9 (9.2%)	10 (5.9%)	11 (7.1%)	− 39.1	< 0.001	< 0.001
High in saturated fats	0 (0.0%)	0 (0.0%)	0 (0.0%)	0 (0.0%)	0.0	0.905	-
High in sodium	2 (1.0%)	0 (0.0%)	1 (0.6%)	0 (0.0%)	− 1.0	0.374	0.301
**Yogurts**	*N* = 150	*N* = 165	*N* = 139	*N* = 127			
Any “high in”^cef^	50 (33.3%)	46 (27.9%)	32 (23.0%)	0 (0.0%)	− 33.3	0.001	< 0.001
High in energy	0 (0.0%)	0 (0.0%)	0 (0.0%)	0 (0.0%)	0	0.934	-
High in sugars^cef^	49 (32.7%)	46 (27.9%)	32 (23.0%)	0 (0.0%)	− 32.7	0.001	< 0.001
High in saturated fats	1 (0.7%)	0 (0.0%)	2 (1.4%)	0 (0.0%)	− 0.7	0.566	0.890
High in sodium	0 (0.0%)	0 (0.0%)	0 (0.0%)	0 (0.0%)	0	0.934	-
**Breakfast cereals**	*N* = 171	*N* = 102	*N* = 128	*N* = 111			
Any “high in”^bcef^	158 (92.4%)	93 (91.2%)	106 (82.8%)	74 (66.7%)	− 25.7	< 0.001	< 0.001
High in energy^bcef^	158 (92.4%)	93 (91.2%)	106 (82.8%)	74 (66.7%)	− 25.7	< 0.001	< 0.001
High in sugars^bcdef^	143 (83.6%)	79 (77.5%)	81 (63.3%)	48 (43.2%)	− 40.4	< 0.001	< 0.001
High in saturated fats^cef^	34 (19.9%)	25 (24.5%)	25 (19.5%)	9 (8.1%)	− 11.8	0.011	0.020
High in sodium	21 (12.3%)	16 (15.7%)	18 (14.1%)	9 (8.1%)	− 4.2	0.292	0.397
**Sweet baked products**	*N* = 180	*N* = 151	*N* = 138	*N* = 148			
Any “high in”	180 (100.0%)	149 (98.7%)	137 (99.3%)	145 (98.0%)	− 2	0.154	0.142
High in energy	179 (99.4%)	149 (98.7%)	137 (99.3%)	145 (98.0%)	− 1.4	0.282	0.359
High in sugars^cf^	177 (98.3%)	142 (94.0%)	136 (98.6%)	135 (91.2%)	− 7.1	0.008	0.045
High in saturated fats^ad^	162 (90.0%)	115 (76.2%)	120 (87.0%)	123 (83.1%)	− 6.9	0.070	0.577
High in sodium^d^	14 (7.8%)	9 (6.0%)	19 (13.8%)	11 (7.4%)	− 0.4	0.922	0.342
**Desserts, ice-creams, and processed fruits**	*N* = 248	*N* = 84	*N* = 101	*N* = 97			
Any “high in”^bcef^	212 (85.5%)	68 (81.0%)	70 (69.3%)	53 (54.6%)	− 30.9	< 0.001	< 0.001
High in energy^abde^	111 (44.8%)	15 (17.9%)	31 (30.7%)	33 (34.0%)	− 10.8	0.073	0.036
High in sugars^bcef^	210 (84.7%)	68 (81.0%)	69 (68.3%)	52 (53.6%)	− 31.1	< 0.001	< 0.001
High in saturated fats	77 (31.0%)	23 (27.4%)	35 (34.7%)	32 (33.0%)	2.0	0.714	0.499
High in sodium	1 (0.4%)	0 (0.0%)	0 (0.0%)	0 (0.0%)	− 0.4	0.919	0.370
**Candies and sweet confectionery**	*N* = 357	*N* = 369	*N* = 377	*N* = 376			
Any “high in”^a^	325 (91.0%)	315 (85.4%)	329 (87.3%)	325 (86.4%)	− 4.6	0.053	0.191
High in energy^a^	322 (90.2%)	314 (85.1%)	326 (86.5%)	325 (86.4%)	− 3.8	0.118	0.280
High in sugars^a^	314 (88.0%)	305 (82.7%)	318 (84.4%)	314 (83.5%)	− 4.5	0.089	0.237
High in saturated fats^bcde^	195 (54.6%)	201 (54.5%)	176 (46.7%)	175 (46.5%)	− 8.1	0.029	0.004
High in sodium	4 (1.1%)	4 (1.1%)	4 (1.1%)	1 (0.3%)	− 0.8	0.221	0.258
**Sweet spreads**	*N* = 119	*N* = 57	*N* = 88	*N* = 73			
Any “high in”^abcde^	109 (91.6%)	43 (75.4%)	39 (44.3%)	33 (45.2%)	− 46.4	< 0.001	< 0.001
High in energy^d^	43 (36.1%)	25 (43.9%)	22 (25.0%)	23 (31.5%)	− 4.6	0.523	0.112
High in sugars^abc^	69 (58.0%)	15 (26.3%)	20 (22.7%)	10 (13.7%)	− 44.3	< 0.001	< 0.001
High in saturated fats^ade^	43 (36.1%)	32 (56.1%)	24 (27.3%)	26 (35.6%)	− 0.5	0.951	0.182
High in sodium	5 (4.2%)	0 (0.0%)	0 (0.0%)	0 (0.0%)	− 4.2	0.189	0.012
**Savory baked products**	*N* = 148	*N* = 121	*N* = 109	*N* = 114			
Any “high in”^bcde^	112 (75.7%)	93 (76.9%)	53 (48.6%)	42 (36.8%)	− 38.9	< 0.001	< 0.001
High in energy^bcde^	103 (69.6%)	92 (76.0%)	52 (47.7%)	42 (36.8%)	− 32.8	< 0.001	< 0.001
High in sugars	4 (2.7%)	6 (5.0%)	0 (0.0%)	0 (0.0%)	− 2.7	0.190	0.014
High in saturated fats^bcde^	29 (19.6%)	34 (28.1%)	10 (9.2%)	8 (7.0%)	− 12.6	0.006	< 0.001
High in sodium^bcdef^	78 (52.7%)	66 (54.5%)	25 (22.9%)	14 (12.3%)	− 40.4	< 0.001	< 0.001
**Nuts and snacks**	*N* = 127	*N* = 110	*N* = 121	*N* = 128			
Any “high in”	122 (96.1%)	108 (98.2%)	118 (97.5%)	125 (97.7%)	1.6	0.494	0.557
High in energy	117 (92.1%)	107 (97.3%)	118 (97.5%)	125 (97.7%)	5.6	0.065	0.034
High in sugars	4 (3.1%)	2 (1.8%)	2 (1.7%)	2 (1.6%)	− 1.5	0.444	0.395
High in saturated fats^bce^	47 (37.0%)	28 (25.5%)	28 (23.1%)	19 (14.8%)	− 22.2	< 0.001	< 0.001
High in sodium^cef^	81 (63.8%)	73 (66.4%)	72 (59.5%)	53 (41.4%)	− 22.4	0.001	< 0.001
**Savory spreads, seasonings, and dressings**	*N* = 274	*N* = 163	*N* = 276	*N* = 278			
Any “high in”^abc^	261 (95.3%)	130 (79.8%)	230 (83.3%)	216 (77.7%)	− 17.6	< 0.001	< 0.001
High in energy^abc^	129 (47.1%)	39 (23.9%)	77 (27.9%)	71 (25.5%)	− 21.6	< 0.001	< 0.001
High in sugars	30 (10.9%)	20 (12.3%)	45 (16.3%)	36 (12.9%)	2.0	0.473	0.227
High in saturated fats^abc^	109 (39.8%)	31 (19.0%)	66 (23.9%)	55 (19.8%)	− 20.0	< 0.001	< 0.001
High in sodium^abc^	238 (86.9%)	123 (75.5%)	215 (77.9%)	195 (70.1%)	− 16.8	< 0.001	< 0.001
**Cheeses**	*N* = 119	*N* = 64	*N* = 79	*N* = 92			
Any “high in”^c^	89 (74.8%)	47 (73.4%)	49 (62.0%)	57 (62.0%)	− 12.8	0.048	0.015
High in energy	16 (13.4%)	10 (15.6%)	10 (12.7%)	7 (7.6%)	− 5.8	0.199	0.195
High in sugars	0 (0.0%)	0 (0.0%)	0 (0.0%)	0 (0.0%)	0.0	0.898	-
High in saturated fats	34 (28.6%)	15 (23.4%)	13 (16.5%)	16 (17.4%)	− 11.2	0.065	0.024
High in sodium	84 (70.6%)	45 (70.3%)	49 (62.0%)	57 (62.0%)	− 8.6	0.189	0.104
**Ready-to-eat meals**	*N* = 140	*N* = 70	*N* = 86	*N* = 80			
Any “high in”^de^	70 (50.0%)	45 (64.3%)	39 (45.3%)	33 (41.3%)	− 8.7	0.216	0.075
High in energy^e^	12 (8.6%)	7 (10.0%)	6 (7.0%)	0 (0.0%)	− 8.6	0.058	0.022
High in sugars	1 (0.7%)	0 (0.0%)	0 (0.0%)	0 (0.0%)	− 0.7	0.738	0.292
High in saturated fats^ac^	21 (15.0%)	20 (28.6%)	19 (22.1%)	24 (30.0%)	15.0	0.009	0.033
High in sodium	66 (47.1%)	38 (54.3%)	38 (44.2%)	31 (38.8%)	− 8.3	0.234	0.161
**Sausages**	*N* = 376	*N* = 150	*N* = 262	*N* = 216			
Any “high in”^cef^	372 (98.9%)	148 (98.7%)	259 (98.9%)	199 (92.1%)	− 6.8	< 0.001	< 0.001
High in energy	125 (33.2%)	49 (32.7%)	82 (31.3%)	82 (38.0%)	4.8	0.245	0.491
High in sugars	0 (0.0%)	0 (0.0%)	0 (0.0%)	0 (0.0%)	0.0	0.782	-
High in saturated fats	220 (58.5%)	94 (62.7%)	153 (58.4%)	134 (62.0%)	3.5	0.404	0.654
High in sodium^cf^	370 (98.4%)	145 (96.7%)	256 (97.7%)	197 (91.2%)	− 7.2	< 0.001	< 0.001
**Non-sausages meat products**	*N* = 356	*N* = 93	*N* = 202	*N* = 180			
Any “high in”^bcde^	215 (60.4%)	55 (59.1%)	75 (37.1%)	61 (33.9%)	− 26.5	< 0.001	< 0.001
High in energy^bcde^	24 (6.7%)	8 (8.6%)	4 (2.0%)	1 (0.6%)	− 6.1	0.010	< 0.001
High in sugars	2 (0.6%)	0 (0.0%)	0 (0.0%)	0 (0.0%)	− 0.6	0.547	0.152
High in saturated fats^de^	98 (27.5%)	35 (37.6%)	49 (24.3%)	43 (23.9%)	− 3.6	0.377	0.162
High in sodium^bcdef^	188 (52.8%)	47 (50.5%)	47 (23.3%)	25 (13.9%)	− 38.9	< 0.001	< 0.001
**Soups**	*N* = 146	*N* = 82	*N* = 65	*N* = 83			
Any “high in”^abc^	146 (100.0%)	75 (91.5%)	56 (86.2%)	72 (86.7%)	− 13.3	0.009	< 0.001
High in energy	0 (0.0%)	0 (0.0%)	0 (0.0%)	0 (0.0%)	0	0.779	-
High in sugars	0 (0.0%)	0 (0.0%)	0 (0.0%)	0 (0.0%)	0	0.779	-
High in saturated fats	0 (0.0%)	0 (0.0%)	0 (0.0%)	0 (0.0%)	0	0.779	-
High in sodium^abc^	146 (100.0%)	75 (91.5%)	56 (86.2%)	72 (86.7%)	− 13.3	0.009	< 0.001

Values represent the sample size and the proportion of regulated products

Cutoffs correspond to the limits on the amount of energy or nutrient of concern for the full implementation of the law (i.e., for solids, per 100 g: 275 kcal of energy, 10 g of sugars, 4 g of saturated fats, 400 mg of sodium; for liquids, per 100 mL: 70 kcal of energy, 5 g of sugars, 3 g of saturated fats, 100 mg of sodium)

T0: preimplementation period, January to February 2015 + January to February 2016 (*n* = 3864); T1: postimplementation of the 1st phase of the law, January to February 2017 (*n* = 2424); T2: postimplementation of the 2nd phase of the law, January to February 2019 (*n* = 3065); T3: postimplementation of the 3rd phase of the law, January to February 2020 (*n* = 2888)

^a^*p*-value < 0.05. comparisons between T0 and T1 were made by contrasting estimated marginal means (EMMs) from Firth’s bias-reduced logistic regression

^b^*p*-value < 0.05. comparisons between T0 and T2 were made by contrasting estimated marginal means (EMMs) from Firth’s bias-reduced logistic regression

^c^*p*-value < 0.05. comparisons between T0 and T3 were made by contrasting estimated marginal means (EMMs) from Firth’s bias-reduced logistic regression

^d^*p*-value < 0.05. comparisons between T1 and T2 were made by contrasting estimated marginal means (EMMs) from Firth’s bias-reduced logistic regression

^e^*p*-value < 0.05. comparisons between T1 and T3 were made by contrasting estimated marginal means (EMMs) from Firth’s bias-reduced logistic regression

^f^*p*-value < 0.05. comparisons between T2 and T3 were made by contrasting estimated marginal means (EMMs) from Firth’s bias-reduced logistic regression

^*^p-value < 0.05. comparisons between T0 and T3 were made by contrasting estimated marginal means (EMMs) from Firth’s bias-reduced logistic regression

^**^p-value for Cochrane Armitage Test for trend

In Table 1, we found that the overall proportion of products with high critical nutrient content significantly decreased from 70.8% prior to the law to 52.5% after full implementation (*p* < 0.001). The largest reductions occurred for “high in” sodium (− 10.6 percentual points (pp)) and “high in” sugars (− 10.5 pp), followed by “high in” saturated fats (− 4.7 pp), and energy (− 3.1 pp). For solid foods, the proportion of “high in” products significantly decreased from 80.5% before the law to 66.8% after the final phase. Among liquids, the proportion of “high in” products decreased from 51.9% before the law to 26.1% after full implementation (*p* < 0.001).

The proportion of products with high content of sugars decreased significantly from pre-law (T0) to the final phase of the law (T3) in all sweet food categories (*p* < 0.05), except for candies and sweet confectionery (Table 2). Substantial decreases (> 30 pp) were observed in the prevalence of “high in” sugars products for sweet spreads, breakfast cereals, milk and milk-based drinks, yogurts, and desserts. Reductions in “high in” sodium products between T0 and T3 were present in all savory categories but did not reach statistical significance in cheeses and ready-to-eat meals (*p* < 0.25). The largest decreases (~ 40 pp) were observed for non-sausage meat products and savory baked products.

Decreases in the proportion of “high in” saturated fats and energy were less consistent. Changes in saturated fats were of smaller magnitude, with significant decreases larger than 20 pp in nuts and snacks and savory spreads and more than 10 pp in savory baked products and breakfast cereals. Importantly, we found increases in the proportion “high in” saturated fats for ready-to-eat-meals (+ 15.0 pp). Decreases in the proportion of “high in” energy products were greater than 20 pp in savory baked products, breakfast cereals, and savory spreads.

With only a few exceptions, the proportion of “high in” products decreased progressively through the different phases of the law with significant *p*-values for trends in almost all nutrients and in all food categories in which we observed significant changes between pre-law and after the final phase (Table 2).

### Changes in the distribution of energy and regulated nutrients

Table 3 presents the quantile regression analyses by food and beverage groups. We observed that in all food and beverage categories, except for ready-to-eat meals, the distribution of at least one regulated nutrient or energy shifted to the left (i.e., the nutrient content decreased and concentrated towards the lower end of the distribution) after the regulation.

**Table 3:** Changes in quartiles of energy and nutrients of concern before (T0) and after each phase of Chile’s Law (T1, T2, T3) for solids and liquids and by food and beverage group, repeated cross-sectional analysis of best-selling items

	**Energy**				**Total sugars**				**Saturated fats**				**Sodium**			
	**(kcal/100 g-mL)**				**(g/100 g-mL)**				**(g/100 g-mL)**				**(mg/100 g-mL)**			
	**2016 (T**_**0**_**)**	**2017 (T**_**1**_**)**	**2019 (T**_**2**_**)**	**2020 (T**_**3**_**)**	**2016 (T**_**0**_**)**	**2017 (T**_**1**_**)**	**2019 (T**_**2**_**)**	**2020 (T**_**3**_**)**	**2016 (T**_**0**_**)**	**2017 (T**_**1**_**)**	**2019 (T**_**2**_**)**	**2020 (T**_**3**_**)**	**2016 (T**_**0**_**)**	**2017 (T**_**1**_**)**	**2019 (T**_**2**_**)**	**2020 (T**_**3**_**)**
**Overall**																
p25	41.0	**37.0**	**32.0**	**33.1**	0.5	0.5	0.5	0.5	0.0	0.0	0.0	0.0	32.0	26.3	**21.0**	27.0
p50	135.0	149.0	122.0	134.0	3.9	**4.7**	3.7	3.8	1.0	0.8	0.8	1.0	164.0	**102.0**	**102.0**	**115.0**
p75	347.0	**390.0**	347.0	362.0	14.0	16.3	**10.5**	**9.9**	6.0	**5.3**	**4.5**	**4.6**	475.0	**397.0**	**398.0**	**388.0**
**Solids**																
p25	118.8	117.0	112.0	**135.0**	0.5	**1.0**	0.5	0.5	0.3	0.1	0.2	0.6	100.0	**57.0**	**73.0**	88.0
p50	275.0	**300.0**	272.0	292.0	3.9	**6.6**	4.0	4.3	3.2	2.8	**2.6**	3.0	340.0	**227.0**	**300.0**	317.0
p75	417.0	**466.0**	430.0	**461.0**	23.3	26.9	21.0	26.0	9.4	9.0	8.3	9.5	641.0	**500.0**	**496.0**	**491.0**
**Liquids**																
p25	12.0	**3.0**	**4.0**	**3.0**	0.4	**0.1**	**0.2**	**0.1**	0.0	0.0	0.0	0.0	8.3	**5.8**	8.0	8.0
p50	32.2	**24.0**	**21.0**	**21.0**	3.8	**2.7**	3.2	**2.9**	0.0	0.0	0.0	0.0	21.2	18.0	18.0	20.0
p75	52.0	**39.0**	**39.0**	**39.0**	8.0	**5.0**	**4.8**	**4.9**	0.2	**0.0**	**0.0**	**0.1**	65.0	60.0	**54.0**	57.0
**Only beverages and milks and milk-based drinks**																
p25	2.0	1.9	2.0	**1.0**	0.1	0.0	**0.0**	**0.0**	0.0	0.0	0.0	0.0	2.2	1.9	2.0	1.4
p50	13.0	12.5	12.0	11.0	2.9	2.6	**2.2**	**2.0**	0.0	0.0	0.0	0.0	9.0	9.0	10.0	**10.0**
p75	38.0	**29.0**	**22.0**	**22.0**	8.0	**5.7**	**4.8**	**4.8**	0.0	0.0	0.0	0.0	16.9	18.0	18.0	18.0
**Other liquids**																
p25	32.0	**24.3**	**27.7**	**24.9**	1.3	**0.5**	1.0	0.7	0.1	0.0	**0.0**	**0.0**	45.0	**51.0**	44.0	44.3
p50	43.0	**35.3**	**37.0**	**36.0**	4.7	3.9	4.6	4.4	0.7	**0.1**	**0.1**	**0.1**	70.0	70.0	**63.0**	65.0
p75	95.0	**54.0**	**55.1**	**54.4**	8.0	**5.0**	**5.0**	**4.9**	2.0	**1.0**	**1.1**	**1.1**	333.6	355.7	268.4	295.0
**Beverages**																
p25	2.0	**1.1**	**1.1**	**1.0**	0.1	0.1	0.0	**0.0**	0.0	0.0	0.0	0.0	3.0	1.1	1.8	1.4
p50	13.0	12.1	**10.0**	11.0	2.9	2.0	**1.8**	**2.0**	0.0	0.0	0.0	0.0	9.0	8.0	10.0	10.0
p75	38.0	**28.0**	**22.0**	**22.0**	8.0	**5.6**	**4.8**	**4.8**	0.0	0.0	0.0	0.0	17.0	18.0	18.0	18.0
**Milks and milk-based drinks**																
p25	36.2	36.0	35.2	36.0	4.8	4.6	**4.6**	4.6	0.1	**0.0**	**0.0**	**0.0**	42.0	**49.0**	43.6	40.0
p50	44.0	43.0	44.0	49.0	5.3	**4.8**	**4.8**	4.9	0.7	0.3	0.3	0.9	51.0	55.0	55.0	54.0
p75	59.0	**54.0**	**52.0**	**52.0**	7.3	**5.0**	**5.0**	**5.0**	1.0	1.0	1.0	1.0	70.0	**61.0**	67.0	**65.0**
**Yogurts**																
p25	49.0	52.0	**53.0**	53.0	5.7	6.9	6.5	6.5	0.0	0.0	0.0	0.1	54.7	54.0	**49.0**	**44.0**
p50	71.0	69.0	74.0	74.0	8.2	8.2	9.6	9.1	0.1	0.2	0.4	**0.7**	55.0	55.0	54.0	**52.0**
p75	90.0	90.0	92.0	85.0	13.1	13.1	**10.0**	**9.9**	1.2	1.2	1.4	**1.7**	65.0	65.0	61.0	**58.0**
**Breakfast cereals**																
p25	368.0	**348.0**	**343.0**	**354.0**	16.5	14.0	**4.7**	**2.7**	1.1	1.2	1.2	1.2	100.0	102.0	105.0	104.0
p50	386.0	**362.0**	**365.0**	**373.0**	22.0	**19.0**	**14.0**	**9.5**	1.9	1.8	1.8	1.8	183.0	190.0	215.0	**275.0**
p75	403.0	391.0	393.0	397.0	30.0	**22.0**	24.0	29.9	3.9	4.0	3.8	3.6	303.0	**374.0**	**350.0**	**377.0**
**Sweet baked products**																
p25	456.0	447.0	453.0	456.0	25.2	**21.4**	26.0	26.0	7.0	**4.5**	6.6	8.0	187.0	188.0	187.0	171.0
p50	484.0	471.0	481.0	484.0	29.5	28.9	**32.0**	**32.7**	10.1	8.5	10.3	11.0	274.0	267.0	260.0	**248.0**
p75	503.0	500.0	499.0	503.0	36.7	35.0	37.0	37.2	12.0	12.0	12.7	13.0	326.0	341.0	330.0	320.0
**Desserts, ice-creams, and processed fruits**																
p25	80.0	81.0	78.0	81.0	13.0	13.3	11.6	**9.9**	0.0	0.0	0.0	0.0	13.5	13.5	10.0	12.0
p50	100.0	107.0	99.0	107.0	15.9	17.1	14.7	14.9	1.1	0.8	0.0	1.3	42.0	48.0	**52.0**	**54.0**
p75	146.0	144.0	148.0	158.0	19.5	20.2	**16.4**	**17.1**	4.1	3.9	4.4	4.3	65.0	77.0	78.0	79.0
**Candies and sweet confectionery**																
p25	342.0	342.0	343.0	340.0	47.0	**43.0**	**43.0**	43.6	0.0	0.0	0.0	0.0	36.0	**20.0**	**23.0**	**23.0**
p50	474.0	474.1	**424.0**	**419.0**	55.0	54.0	54.0	55.0	9.4	9.8	**4.7**	**4.0**	84.0	68.0	72.0	72.0
p75	541.0	540.0	539.0	530.0	64.0	64.0	66.0	65.0	17.0	17.7	16.5	16.0	142.0	125.0	125.0	124.0
**Sweet spreads**																
p25	100.6	81.5	**29.0**	**28.2**	3.7	3.8	3.9	3.6	0.0	0.0	0.0	0.0	15.0	**25.0**	12.9	14.0
p50	169.0	228.0	**56.0**	**58.0**	8.1	6.0	6.2	4.8	0.2	6.0	0.0	0.1	27.0	37.0	21.0	21.0
p75	317.0	317.0	227.0	238.0	35.7	14.2	**8.8**	**7.2**	13.5	18.8	8.8	13.0	53.8	55.1	**39.8**	**40.0**
**Savory baked products**																
p25	271.0	279.0	**257.0**	**255.0**	1.7	2.0	**2.5**	**2.9**	0.8	0.8	0.9	0.9	381.0	384.0	348.0	352.0
p50	300.0	314.0	280.0	273.0	3.4	3.8	4.1	4.1	1.4	1.5	1.5	1.3	416.0	425.0	**382.0**	**382.0**
p75	408.0	424.0	399.0	404.0	5.1	**6.1**	5.8	5.3	3.5	4.5	2.9	2.9	581.0	591.0	**399.0**	**394.0**
**Nuts and snacks**																
p25	488.0	482.0	494.0	492.0	0.6	1.2	1.0	1.5	3.0	2.9	3.0	2.9	347.0	353.0	354.0	329.0
p50	520.0	510.0	519.0	**508.0**	2.8	2.4	2.8	2.8	4.0	3.6	**3.6**	**3.2**	500.0	488.0	**448.0**	**388.0**
p75	548.0	542.0	548.0	533.0	5.8	5.5	5.5	4.6	6.0	**4.1**	**4.1**	**3.7**	600.0	592.0	**492.0**	**470.0**
**Savory spreads, seasonings, and dressings**																
p25	53.0	46.0	47.0	**36.0**	0.3	0.3	0.4	0.3	0.0	0.0	0.0	0.0	461.0	**399.0**	**399.0**	**380.0**
p50	213.0	**96.0**	**104.0**	**97.0**	1.8	2.2	**2.6**	2.3	2.4	**0.1**	**0.1**	**0.1**	660.0	571.0	647.0	671.0
p75	391.0	**230.0**	**306.0**	**267.3**	4.7	5.7	5.8	6.0	16.5	**3.9**	**4.2**	**3.6**	1079.0	**761.0**	1231.0	1231.0
**Cheeses**																
p25	260.0	260.0	221.0	238.0	0.0	0.0	0.0	0.0	12.8	12.8	12.4	12.0	384.0	364.0	342.0	360.0
p50	300.0	305.0	314.0	292.0	0.2	0.3	0.2	0.3	14.6	15.2	15.4	15.2	556.0	561.0	480.0	496.0
p75	350.0	343.0	344.0	340.0	1.9	1.9	1.9	1.3	18.3	**17.3**	17.9	17.7	995.0	770.0	**662.0**	815.0
**Ready-to-eat meals**																
p25	66.0	33.0	79.0	100.0	0.5	0.7	**1.0**	0.7	0.0	0.0	0.0	0.0	255.2	**300.0**	**294.0**	**308.0**
p50	118.8	135.0	136.1	**150.3**	1.2	1.3	**1.8**	**1.7**	0.3	0.8	1.0	1.7	390.0	431.0	371.0	370.0
p75	200.0	**258.0**	250.0	221.0	2.0	2.0	**3.2**	**3.2**	2.6	4.3	3.6	**4.3**	504.0	**620.0**	564.0	549.0
**Sausages**																
p25	127.0	120.0	123.0	128.0	0.2	**0.5**	**0.5**	**0.5**	2.9	1.8	2.0	3.6	800.0	**730.0**	**677.0**	**677.0**
p50	227.0	235.0	204.0	234.0	0.5	0.5	0.5	0.5	7.0	6.2	**5.1**	7.0	899.0	**789.0**	**790.0**	**790.0**
p75	296.0	283.0	294.0	311.0	1.4	**0.8**	**0.5**	**0.5**	10.0	9.6	9.5	10.3	1072.0	991.0	**950.0**	**966.0**
**Non-sausages meat products**																
p25	117.0	125.0	108.0	117.0	0.0	0.0	0.0	0.0	1.1	**1.6**	0.8	0.9	300.0	273.0	**204.0**	299.0
p50	160.0	**184.0**	154.0	156.0	0.0	**0.5**	0.0	**0.5**	2.1	**3.1**	1.8	1.8	413.0	403.0	**349.0**	**350.0**
p75	218.0	**243.0**	210.0	218.0	0.5	0.6	0.5	0.5	4.7	5.9	4.5	4.5	559.0	600.0	**400.0**	**388.0**
**Soups**																
p25	23.4	23.5	23.9	**22.3**	0.3	0.3	0.3	0.3	0.0	0.1	0.0	0.0	304.7	272.9	265.0	257.8
p50	25.6	25.8	26.5	25.3	0.6	0.7	0.7	0.7	0.1	0.1	0.1	0.1	344.9	329.2	310.6	**303.3**
p75	33.1	32.9	32.1	30.2	1.4	1.5	1.3	1.4	0.3	0.4	0.2	0.2	386.6	376.7	374.4	375.3

T0: preimplementation period, January to February 2015 + January to February 2016 (*n* = 3864); T1: postimplementation of the 1st phase of the law, January to February 2017 (*n* = 2424); T2: postimplementation of the 2nd phase of the law, January to February 2019 (*n* = 3065); T3: postimplementation of the 3rd phase of the law, January to February 2020 (*n* = 2888)

Quartiles and *p*-values were obtained from quantile regression models (one model per nutrient per food or beverage group), using implementation period as independent variable. Significant *p*-values are bold and represent a *p*-value < 0.05 versus T0

We found improvements in total sugars distribution across most sweet food and beverage groups (T3 vs T0). Significant reductions were observed at the 50th or 75th percentile for beverages, milk and milk-based drinks, yogurts, breakfast cereals, desserts, ice creams, and processed fruits, and sweet spreads, with decreases ranging from − 0.9 to − 28.5 g of sugars/100 g or mL, depending on the food group. In contrast, there were no reductions in sweet baked products and candies and sweet confectionery,

We also found improvements in the sodium distributions for savory food groups (T3 vs T0). Decreases were significant in the 50th or 75th percentile in most savory food categories, except for cheeses, ready-to-eat meals, and savory spreads, seasonings, and dressings. The reductions ranged from − 34.0 to − 187.0 mg of sodium/100 g or mL, depending on the food group. For sausages, there was an improvement across the entire distribution.

For saturated fats, we observed improvements in nuts and snacks and savory spreads, seasonings, and dressings, and candies in the 50th or 75th percentile, with reductions ranging from − 0.8 to − 12.9 g of saturated fats per 100 g (T3 vs T0). However, the content of saturated fats slightly increased in yogurts and ready-to-eat meals.

Energy distributions remained similar in most food and beverage categories with smaller improvements in some categories (T3 vs T0); the largest improvements were observed in savory spreads (75th percentile changed from 391.0 to 267.3 kcal per 100 g), sweet spreads (50th percentile changed from 169.0 to 58.0 kcal per 100 g) and beverages (75th percentile changed from 38.0 to 22.0 kcal per 100 g). Nonetheless, we also found that energy content increased in ready-to-eat meals (50th percentile changed from 118.8 to 150.3 kcal per 100 g).

**Fig. 3:**
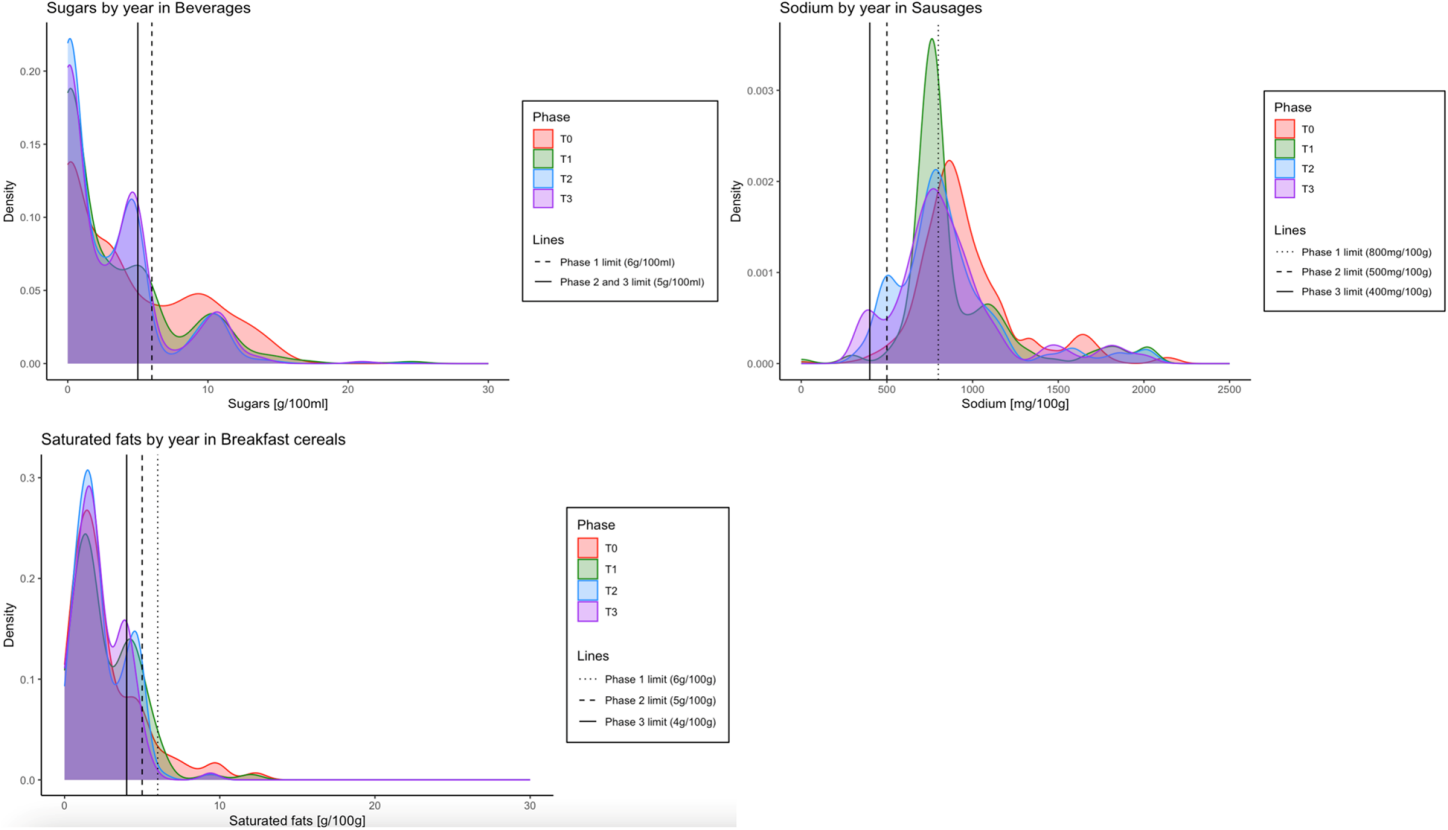
Density curves for the amounts of total sugars in beverages, sodium in sausages, and saturated fats in breakfast cereals, repeated cross-sectional analysis of best-selling items. The red line represents the distribution in T0 (preimplementation period, January to February 2015 + January to February 2016); the green line represents the distribution in T1 (postimplementation of the 1st phase of the law, January to February 2017); the blue line represents the distribution in T2 (postimplementation of the 2nd phase of the law, January to February 2019); and the purple line represents the distribution in T3 (postimplementation of the 3rd phase of the law, January to February 2020)

All the changes observed in the distribution were aligned with the increasingly stricter limits of the law (see examples for sugars, sodium, and saturated fats in Fig. 3).

### Sensitivity analyses

In the full sample, the direction and significance of the changes remained consistent (Additional File 1: Tables S2-S4). However, we observed that the proportion of “high in” products is similar in liquids but of lower magnitude in solids, except for energy. This results in an overall smaller decrease in the magnitude of changes in energy, sugars, and sodium. Analyses by food categories confirm that the direction and significance of changes remain the same overall, though the magnitude is smaller for solid foods.

## Discussion

To our knowledge, this is the first study examining the association between the full implementation of this innovative set of policies and changes both in the proportion of regulated products and the content of regulated nutrients in the food supply. We found that the proportion of “high in” products (as defined in the third phase of the law) decreased substantially throughout the different phases, especially for products with high sugars and sodium content. Analyses of the changes in the distribution showed left shifts in the contents of sodium and sugars. In contrast, the decreases in saturated fats and energy were less frequent and of smaller magnitude. Overall, we observed that almost all foods and beverage categories showed improvements in the distribution of at least one nutrient of concern.

The Chilean law was implemented in three phases in which the limits of the regulation became increasingly stricter. This stepped implementation was an agreement between the government and the food industry to provide enough time for product reformulation [1]. Our analyses indicate that this strategy may have contributed to decreases in the proportion of “high in” products, and the progressive left shifts in the distribution of critical nutrients throughout the regulation phases. Importantly, these results also highlight the need for statutory policies to promote improvements in the food supply. Previous research showed that before the Chilean labelling law, there were almost no changes in the packaged food supply [11]. Here, we show that increasingly stricter cutoffs were associated with changes. This evidence contrasts with the subtle changes found in voluntary policies and highlights the relevance of making regulations mandatory [13].

There are only a few examples of the effectiveness of front-of-package warning label policies in reformulation. After the initial implementation of the Chilean law, there was a reduction of 7 percentage points (from 51 to 44%, *p* < 0.001) in the proportion of regulated products, mainly in the case of “high in” sugars and sodium products [6]. Our results align with those reported after the initial implementation of the Chilean law. When comparing the initial implementation using the stricter nutrient limits for the final phase of the law, we observed reductions of 8 percentage points (from 70.8 to 63.4%). The decrease is even larger when we compare the proportion of “high in” products after the full implementation of the law. Compared to the baseline, we found a decrease of 17.8 percentage points (from 70.8 to 52.5%), suggesting that the food industry continued reformulating products. Our results also align with an analysis of changes in “high in” products in Peru, the second country to implement a front-of-package warning label law in Latin America. The Peruvian study shows that the proportion of “high in” foods and beverages decreased after 2 years of the regulation’s initial implementation, mostly due to the decrease in the proportion of “high in” beverages [14]. Unfortunately, we cannot compare the magnitude of the changes between the two studies because of differences in methodological approaches and sample sizes.

In the final phase of the law, the largest changes occurred in the proportion of regulated products with high sugars and sodium contents, while changes for products with high energy and saturated fats contents were less frequent and of smaller magnitude. For “high in” sugars, the group with the largest decrease was sweet spreads (from 58.0 to 13.7%, *p* < 0.05), followed by breakfast cereals and milk and milk-based drinks. For “high in” sodium, the group with the biggest decrease was savory baked products (from 52.8 to 13.9%, *p* < 0.05), followed by non-sausage meat products and nuts and snacks. We also found that the food categories showing reductions in sugars and sodium were those in which the regulated nutrient content before the law was closer to the law’s limits. On the other hand, the food categories with products containing very high contents of regulated nutrients were not reformulated. One example is the lack of changes in the proportion of “high in” sugars products or the sugars content in sweet baked products and candies and sweet confectionery [6]. These groups were likely not reformulated because their sugars content was considerably higher than the law’s limit of 10 g of sugars per 100 g of product. For instance, the 75th percentile values for these food groups after full implementation of the law were 37.2 and 65.0 g of sugars per 100 g, respectively.

Regarding the decreases in sugars, this might be explained by the possibility of replacing sugars with nonnutritive sweeteners (NNS) without affecting the overall sweetness of the beverages and foods. In fact, a previous study found that after the initial implementation of the law, approximately 34% of the products that reformulated their sugars content started using at least one NNS [15]. However, we have reported that despite the increase in NNS use, the overall sweetness of the beverages purchased did not vary [16]. Notably, we found that in sweet baked products, changes in total sugars content were of smaller magnitude compared to other beverage or food categories, likely because of the challenges associated with achieving the sweetness but also the bulking, browning, and other properties that sucrose, glucose, and fructose provide in these products [17].

We also observed important reductions and changes in the distribution of sodium in most savory food groups, especially among sausages, non-sausage meat products, savory baked products, and nuts and snacks. Notably, at the initial implementation of the law, the changes in sodium content were mostly around the 25th or 50th percentile [6]. After the implementation of the final phase, we found larger left shifts that applied to the entire sodium distribution. It is unclear whether sodium was replaced by salt substitutes. Further analyses should assess whether alternative salt substitutes such as potassium chloride, magnesium chloride, or monosodium glutamate were used to compensate for sodium declines [18].

After the initial implementation of the Chilean law, changes in saturated fats were minimal [6]. In the final phase of this policy, we found that the proportion of “high in” saturated fats products decreased by 4.7 percentage points, a decrease of smaller magnitude compared to sugars or sodium. In Peru, there was also a small reduction in saturated fats after implementing a similar labelling law [14]. In contrast, a limited evaluation of reformulation after incorporating the Choices nutritional logo showed significant decreases in the saturated fats content of dairy products [19]. One potential explanation for the minimal changes in saturated fats is their addition to foods as components of food-based ingredients (e.g., oils, milk), where saturated fats cannot be easily removed. Another explication is the technical challenge related to melting point and oxidation, which have been described as barriers to improving the saturated fats composition of the food supply [20, 21].

We also found that the overall proportion of “high in” energy products decreased significantly, but only by 3.1 percentage points. Additionally, the reductions “high in” energy were significant for less than half of the analyzed food groups, while the other groups showed no change or even small, non-significant increases. It is unclear why we do not see widespread changes in energy, but it seems that most of the food groups that did not change in energy, such as sweet baked products, candies and sweet confectionery, and sausages, were not reformulated in sugars or saturated fats either.

Interestingly, we also observed that in some food categories, such as breakfast cereals, reductions in sugars left shifts were accompanied by increases in sodium. This finding suggests that producers try to maximize flavors, as taste-taste interactions have been described and are currently a potential strategy for decreasing the critical nutrient content of foods [18, 22, 23].

Our sensitivity analyses of the entire food supply confirm the direction of the reported results; however, they suggest a lower change magnitude. We have previously reported that after the implementation of the regulation there was a decrease in the purchases of “high in” products [5, 24]. Thus, differences between the best-selling food categories and the total sample could be explained because consumers purchased more reformulated products. Moreover, the total sample included international products that are less likely to be reformulated because of the regulation of a small market such as Chile. The total sample also included products from small and very small industries that had a three-year delay in implementing the law [1]. Nonetheless, to fully understand these differences, further analyses should be conducted with this specific objective.

Improving the quality of the food supply has been suggested as a way to address the ongoing pandemic of NCDs [25]. Several reports conclude that reformulation is a cost-effective way of improving people’s diets [26, 27]. Furthermore, reformulation policies are more equitable than those aimed at changing consumer behavior. These policies benefit individuals from low socioeconomic backgrounds, who may face challenges in altering their health behaviors despite receiving additional information [28, 29]. However, the limits of reformulation policies on achieving healthier diets have also been noted [30, 31]. As shown in this study, observed decreases in regulated nutrients could be directly aligned with the regulation limits. Therefore, to observe significant changes, regulations need to be strict enough. More importantly, the sources of nutrients of concern in packaged products may be replaced with other highly processed ingredients and additives rather than shifting diets towards whole or minimally processed foods. For example, in Chile, there was a significant increase in the use of NNS in the food supply after the initial implementation of the law [15]. Furthermore, some authors have even challenged whether some nutrients, such as saturated fats, might be detrimental to human health [32]. Several authors have proposed assessing dietary quality based on food and dietary-based models rather than nutrient profile models [31, 33]. Thus, the ultimate goal of policies should be promoting the intake of natural foods and beverages rather than exclusively reducing the critical nutrients of packaged foods.

Despite the increasing focus on food reformulation to promote healthier diets, there is limited evidence on how food reformulation impacts dietary intake and, ultimately, health outcomes. We have reported that food categories in which we observe reductions in critical nutrients account for approximately 15% of the calories, 40% of added sugars, and 15% of sodium intake of the adult population [34, 35]. In children, the relevance of the reformulated food categories was larger, reaching 25–30% for energy, 60% of added sugars, and 20% of sodium intake [36]. A recent review of modelling studies assessing the impact of food reformulation on dietary intake and health concludes that interventions to reduce sodium result in declines in sodium intake, reductions in cardiovascular disease mortality, and gains in quality-adjusted life years (QALYs) [37]. For sugars, most of the evidence is based on sugars reductions in sugar-sweetened beverages, and it shows that reformulation would reduce energy and sugars intake and decrease both obesity and diabetes; there was no information regarding improvements in quality of life. For saturated fats, evidence precluded arriving at any conclusion [37]. Nonetheless, it is important to note that modelling studies must simplify complex interventions and rely on several assumptions that do not necessarily reflect real-life behaviors. Consumers might react to reformulation by changing to other non-reformulated products, adding critical nutrients to foods (i.e., salt or table sugar), or increasing the amounts of food consumed. Thus, the actual impact of reformulation on diet and health indicators must come from real-life evaluations.

Our study is not exempt from limitations. The main limitation is the use of observational data and descriptive analyses to assess the changes in the proportion of regulated products and shifts in energy and nutrients of concern after the three phases of the Chilean law. Therefore, our study design does not allow us to establish the causality of the findings. While the trend analysis shows associations over time, it cannot isolate the potential effects of the staggered policy implementation from other factors, such as changes in the supply chain, delayed influences of earlier policy phases, or broader market trends. In observational, non-experimental studies, *p*-values should be interpreted carefully, as they do not imply causality or account for unmeasured confounders. Future quasi-experimental studies, such as controlled interrupted time-series analyses or experimental approaches, could better disentangle the effects of staggered policy implementation. Despite this limitation, our findings provide valuable insights about the changes in the prevalence of “high in” products over time and shifts in the content of regulated nutrients. These changes, which coincide with the progressively stricter limits of the phases of the law, may be influenced by the implementation of the labelling law. A second limitation is that our analyses were conducted on repeated cross-sectional sample, focusing on changes within product categories rather than conducting longitudinal analyses to track the reformulation of the same products over time. As a result, we cannot fully rule out the possibility that our results are influenced by sampling differences. Moreover, we could not differentiate whether changes are due to the reformulation of pre-existing products, the discontinuation of old products, or the introduction of new products on the market. These dynamics can obscure the relative contributions of reformulation versus new product development to observed changes in “high in” product prevalence. However, to make our analytical sample more consistent, we included only foods and beverages representing the best-selling product within each specific category. Another limitation is that we were unable to differentiate which of the different components of the regulation (i.e., labelling, marketing restrictions, or healthier school environments) contributed the most to reformulation. However, we have previously suggested that dietary improvements will derive from multiple-component policies such as the Labelling Law rather than from single policies [38]. Additionally, we focused on the reformulation of regulated nutrients of concern, and we were unable to determine if these nutrients were replaced with other nutrients or additives, as this was outside the scope of our research. Thus, we cannot really assess the overall quality of the food supply. Finally, our analyses are based on information available on nutritional labels; yet, in all the rounds, data were collected similarly, and thus, the error is systematic. Also, data were collected prospectively and directly by research assistants rather than relying on food retailers’ information or retrospective databases; this allowed us to identify changes in the food supply with enough precision to be linked to the different phases of the regulation.

## Conclusions

In conclusion, we have shown that after implementing the final phase of a multi-component policy, such as the Chilean labelling law, there were important reductions in the critical nutrient content of packaged foods, particularly with respect to sodium in solid foods and sugars in beverages. The magnitude of these changes increased as the limits of the regulation became stricter, aligning with the implementation of the regulation. It is important to link these changes in the food supply to changes in the overall dietary quality of the population to fully assess the impact of the regulation.

## Supplementary Information

Additional file 1: Table S1, Figs. S1-S3, Tables S2-S4. Table S1-Market Share of Best-Selling Products by Euromonitor International’s Passport Database. Fig. S1-Front-of-Package Warning Labels Under Chile’s Food Labeling Law. Fig. S2-Product Exclusion Flow Chart for Sensitivity Analyses. Table S2-Changes in "High In" Proportions for Solids and Liquids Across Chile’s Law Phases in the Full Food Supply. Table S3- “High In” Proportions by Food Group Across Chile’s Law Phases in the Full Food Supply. Table S4-Quartile Changes in Energy and Nutrients of Concern Across Chile’s Law Phases in the Full Food Supply.

## Data Availability

The datasets used and/or analyzed during the current study are available from the corresponding author upon reasonable request.

## Abbreviations

ASACH: Chilean National Association of Supermarkets
NCDs: Nutrition-related chronic diseases
NFP: Chilean Nutrition Facts Panel database
NNS: Nonnutritive sweeteners
PP: Percentual points
QALYs: Quality-adjusted life years

## References

[1] Corvalán C, Reyes M, Garmendia ML, Uauy R. Structural responses to the obesity and non-communicable diseases epidemic: update on the Chilean law of food labelling and advertising. Obes Rev. 2019;20(3):367–74. 30549191 10.1111/obr.12802

[2] Correa T, Fierro C, Reyes M, Dillman Carpentier FR, Taillie LS, Corvalan C. Responses to the Chilean law of food labeling and advertising: exploring knowledge, perceptions and behaviors of mothers of young children. Int J Behav Nutr Phys Act. 2019;16(1):21. 30760273 10.1186/s12966-019-0781-x PMC6375144

[3] Mediano Stoltze F, Reyes M, Smith TL, Correa T, Corvalán C, Carpentier FRD. Prevalence of child-directed marketing on breakfast cereal packages before and after Chile’s food marketing law: a pre- and post-quantitative content analysis. Int J Environ Res Public Health. 2019;16(22):4501. 31731577 10.3390/ijerph16224501 PMC6888536

[4] Taillie LS, Reyes M, Colchero MA, Popkin B, Corvalán C. An evaluation of Chile’s Law of Food Labeling and Advertising on sugar-sweetened beverage purchases from 2015 to 2017: a before-and-after study. PLoS Med. 2020;17(2): e1003015. 32045424 10.1371/journal.pmed.1003015 PMC7012389

[5] Taillie LS, Bercholz M, Popkin B, Reyes M, Colchero MA, Corvalán C. Changes in food purchases after the Chilean policies on food labelling, marketing, and sales in schools: a before and after study. Lancet Planet Health. 2021;5(8):e526–33. 34390670 10.1016/S2542-5196(21)00172-8 PMC8364623

[6] Reyes M, Smith Taillie L, Popkin B, Kanter R, Vandevijvere S, Corvalán C. Changes in the amount of nutrient of packaged foods and beverages after the initial implementation of the Chilean Law of Food Labelling and Advertising: a nonexperimental prospective study. PLoS Med. 2020;17(7):e1003220. 32722710 10.1371/journal.pmed.1003220 PMC7386631

[7] Euromonitor International. Euromonitor International Database 2022 [Available from: https://www.euromonitor.com/].

[8] Ministry of Health of Chile. Manual de Etiquetado Nutricional de Alimentos. Nutrition and Foods Department; 2022. https://saludresponde.minsal.cl/wpcontent/uploads/2019/06/2019.06.26_MANUAL-DE-ETIQUETADO_ACTUALIZADO-2019.pdf.

[9] Kanter R, Reyes M, Corvalán C. Photographic methods for measuring packaged food and beverage products in supermarkets. Curr Dev Nutr. 2017;1(10):e001016-e. 29955678 10.3945/cdn.117.001016 PMC5998779

[10] Ministry of Health of Chile. Reglamento Sanitario de los Alimentos. Decreto N° 977/96. Chile; 2022. https://www.minsal.cl/wpcontent/uploads/2015/10/DECRETO_977_96_actualizado_-mayo-2024.pdf.

[11] Kanter R, Reyes M, Vandevijvere S, Swinburn B, Corvalan C. Anticipatory effects of the implementation of the Chilean Law of Food Labeling and Advertising on food and beverage product reformulation. Obes Rev. 2019;20(suppl 2):129–40. 10.1111/obr.12870 31245920

[12] Firth D. Bias reduction of maximum likelihood estimates. Biometrika. 1993;80(1):27–38.

[13] Vandevijvere S, Vanderlee L. Effect of formulation, labelling, and taxation policies on the nutritional quality of the food supply. Curr Nutr Rep. 2019;8(3):240–9. 31321705 10.1007/s13668-019-00289-x

[14] Saavedra-Garcia L, Meza-Hernández M, Diez-Canseco F, Taillie LS. Reformulation of top-selling processed and ultra-processed foods and beverages in the Peruvian food supply after front-of-package warning label policy. Int J Environ Res Public Health. 2023;20(1):424. 10.3390/ijerph20010424 PMC9819345 36612748

[15] Zancheta Ricardo C, Corvalán C, Smith Taillie L, Quitral V, Reyes M. Changes in the use of non-nutritive sweeteners in the Chilean food and beverage supply after the implementation of the food labeling and advertising law. Front Nutr. 2021;8:773450. 10.3389/fnut.2021.773450 PMC8630583 34859036

[16] Rebolledo N, Bercholz M, Corvalán C, Ng SW, Taillie LS. Did the sweetness of beverages change with the Chilean Food Labeling and Marketing Law? A before and after study. Front Nutr. 2022;9:1043665. 10.3389/fnut.2022.1043665 PMC9650246 36386952

[17] Erickson S, Carr J. The technological challenges of reducing the sugar content of foods. Nutr Bull. 2020;45(3):309–14.

[18] Nurmilah S, Cahyana Y, Utama GL, Aït-Kaddour A. Strategies to reduce salt content and its effect on food characteristics and acceptance: a review. Foods. 2022;11(19):3120. 36230196 10.3390/foods11193120 PMC9564303

[19] Vyth EL, Steenhuis IHM, Roodenburg AJC, Brug J, Seidell JC. Front-of-pack nutrition label stimulates healthier product development: a quantitative analysis. Int J Behav Nutr Phys Act. 2010;7(1):65. 20825645 10.1186/1479-5868-7-65 PMC2945986

[20] Fanzo J, McLaren R, Bellows A, Carducci B. Challenges and opportunities for increasing the effectiveness of food reformulation and fortification to improve dietary and nutrition outcomes. Food Policy. 2023;119:102515.

[21] Talbot G. 1 - Saturated fats in foods and strategies for their replacement: an introduction. In: Talbot G, editor. Reducing Saturated Fats in Foods: Woodhead Publishing; 2011. p. 3–28. https://shop.elsevier.com/books/reducing-saturated-fats-in-foods/talbot/978-1-84569-740-2.

[22] Keast RSJ, Breslin PAS. An overview of binary taste–taste interactions. Food Qual Prefer. 2003;14(2):111–24.

[23] Torrico DD, Prinyawiwatkul W. Psychophysical effects of increasing oil concentrations on saltiness and bitterness perception of oil-in-water emulsions. J Food Sci. 2015;80(8):S1885–92. 26199098 10.1111/1750-3841.12945

[24] Taillie LS, Bercholz M, Popkin B, Rebolledo N, Reyes M, Corvalán C. Decreases in purchases of energy, sodium, sugar, and saturated fat 3 years after implementation of the Chilean food labeling and marketing law: an interrupted time series analysis. PLoS Med. 2024;21(9):e1004463. 39331649 10.1371/journal.pmed.1004463 PMC11432892

[25] World Health Organization (WHO). Global action plan for the prevention and control of noncommunicable diseases 2013–2020. Geneva, Switzerland.: World Health Organization; 2013. https://www.who.int/publications/i/item/9789241506236.

[26] Organisation for Economic Co-operation and Development (OECD). The heavy burden of obesity: the economics of prevention. Paris: OECD Health Policy Studies; 2019. https://www.oecd.org/content/dam/oecd/en/publications/reports/2019/10/the-heavy-burden-ofobesity_beeff1b/67450d67-en.pdf.

[27] Mantilla Herrera AM, Crino M, Erskine HE, Sacks G, Ananthapavan J, Mhurchu CN, et al. Cost-effectiveness of product reformulation in response to the health star rating food labelling system in Australia. Nutrients. 2018;10(5):614. 10.3390/nu10050614 PMC5986494 29757979

[28] Griffith R, O’Connell M, Smith K. The importance of product reformulation versus consumer choice in improving diet quality. Economica. 2017;84(333):34–53.

[29] Løvhaug AL, Granheim SI, Djojosoeparto SK, Harrington JM, Kamphuis CBM, Poelman MP, et al. The potential of food environment policies to reduce socioeconomic inequalities in diets and to improve healthy diets among lower socioeconomic groups: an umbrella review. BMC Public Health. 2022;22(1):433. 35246074 10.1186/s12889-022-12827-4 PMC8895543

[30] Scrinis G, Monteiro CA. Ultra-processed foods and the limits of product reformulation. Public Health Nutr. 2017;21(1):247–52. 28703086 10.1017/S1368980017001392 PMC10261094

[31] Dickie S, Woods JL, Baker P, Elizabeth L, Lawrence MA. Evaluating nutrient-based indices against food- and diet-based indices to assess the health potential of foods: how does the Australian Health Star Rating system perform after five years? Nutrients. 2020;12(5):1463. 32443570 10.3390/nu12051463 PMC7284529

[32] de Oliveira Otto MC, Mozaffarian D, Kromhout D, Bertoni AG, Sibley CT, Jacobs DR Jr, et al. Dietary intake of saturated fat by food source and incident cardiovascular disease: the Multi-Ethnic Study of Atherosclerosis. Am J Clin Nutr. 2012;96(2):397–404. 22760560 10.3945/ajcn.112.037770 PMC3396447

[33] Monteiro CA, Cannon G, Levy R, Moubarac J-C, Jaime P, Martins AP, et al. NOVA The star shines bright. World Nutrition. 2016;7(1–3):28–38.

[34] Cediel G, Reyes M, da Costa Louzada ML, Martinez Steele E, Monteiro CA, Corvalan C, et al. Ultra-processed foods and added sugars in the Chilean diet (2010). Public Health Nutr. 2018;21(1):125–33. 28625223 10.1017/S1368980017001161 PMC10260868

[35] Agostini C, Corvalán C, Cuadrado C, Martínez C, Paraje G. Evaluación y Aplicación de Impuestos a los Alimentos con Nutrientes Dañinos para la Salud en Chile. Final Report from the Expert Committee. Santiago, Chile: Ministry of Finance, Ministry of Health and Inter-American Development Bank (IADB); 2018. https://www.df.cl/noticias/site/docs/20180921/20180921171107/2018_03_02_evaluacion_y_aplicacion_de_impuestos__1_.pdf.

[36] Araya C, Corvalan C, Cediel G, Taillie LS, Reyes M. Ultra-processed food consumption among Chilean preschoolers is associated with diets promoting non-communicable diseases. Front Nutr. 2021;8:601526. 33842518 10.3389/fnut.2021.601526 PMC8032866

[37] Federici C, Detzel P, Petracca F, Dainelli L, Fattore G. The impact of food reformulation on nutrient intakes and health, a systematic review of modelling studies. BMC Nutr. 2019;5(1):2. 32153917 10.1186/s40795-018-0263-6 PMC7050744

[38] Popkin BM, Barquera S, Corvalan C, Hofman KJ, Monteiro C, Ng SW, et al. Towards unified and impactful policies to reduce ultra-processed food consumption and promote healthier eating. Lancet Diabetes Endocrinol. 2021;9(7):462–70. 33865500 10.1016/S2213-8587(21)00078-4 PMC8217149

