# Supplementary material for: Changes in the critical nutrient content of packaged foods and beverages after the full implementation of the Chilean Food Labelling and Advertising Law: a repeated cross-sectional study
Source: BMC Med. 2025 Jan 27;23:46. doi: 10.1186/s12916-025-03878-6 (PMC11773852; doi:10.1186/s12916-025-03878-6)
Supplement: Supplementary file 1 — Additional file 1: Table S1, Figs. S1-S3, Tables S2-S4. Table S1-Market Share of Best-Selling Products by Euromonitor International’s Passport Database. Fig. S1-Front-of-Package Warning Labels Under Chile’s Food Labeling Law. Fig. S2-Product Exclusion Flow Chart for Sensitivity Analyses. Table S2-Changes in "High In" Proportions for Solids and Liquids Across Chile’s Law Phases in the Full Food Supply. Table S3- “High In” Proportions by Food Group Across Chile’s Law Phases in the Full Food Supply. Table S4-Quartile Changes in Energy and Nutrients of Concern Across Chile’s Law Phases in the Full Food Supply. [file 12916_2025_3878_MOESM1_ESM.docx]

**Additional file 1. Sensibility analysis results.**

BMC Medicine

Title: Changes in the Critical Nutrient Content of Packaged Foods and Beverages after the Full Implementation of the Chilean Food Labelling and Advertising Law: A repeated cross-sectional study.

**Table S1.** Market share of best-selling products according to Euromonitor International’s Passport database.

We used sales data from Euromonitor International’s Passport database from 2015 to 2020 to include in the analytic sample the best-selling products (i.e., those with >1% market share) within each of the 32 main food groups from the Euromonitor database. The products were selected using the following formula, using the unit in Chilean Pesos (CLP) million:

Market share =

$\frac{Sales of <product or brand family of products from Euromonitor food gruoup> during <year>}{Total of sales of <Euromonitor food group>during <year>} \times100$

The selected brands represented approximately 95% of the market share. However, chocolate confectionery and sweet biscuits, snack bars, and fruit snacks had a lower market share of around 80%.

| **Euromonitor category** | **Percentage of market share** | | | | |
| --- | --- | --- | --- | --- | --- |
|  | **2015** | **2016** | **2017** | **2019** | **2020** |
| **Hot Drinks** |  |  |  |  |  |
| Coffee | 93.5 | 95.1 | 96.0 | 96.7 | 96.5 |
| Tea | 98.1 | 98.2 | 98.1 | 98.2 | 98.3 |
| Other Hot Drinks | 96.5 | 97.3 | 98.0 | 98.9 | 98.2 |
| **Cooking Ingredients and Meals** |  |  |  |  |  |
| Edible Oils | 97.2 | 97.6 | 98.1 | 96.8 | 97.6 |
| Ready Meals | 95.7 | 95.8 | 96.0 | 96.3 | 96.3 |
| Sauces, Dressings and Condiments | 91.1 | 92.9 | 92.8 | 94.0 | 93.9 |
| Soup | 99.5 | 99.5 | 99.5 | 98.4 | 98.7 |
| Sweet Spreads | 99.0 | 97.0 | 99.6 | 99.3 | 99.1 |
| **Dairy Products and Alternatives** |  |  |  |  |  |
| Baby Food | 98.7 | 97.4 | 98.4 | 98.5 | 98.7 |
| Butter and Spreads | 98.5 | 96.6 | 97.4 | 97.9 | 98.1 |
| Cheese | 96.9 | 96.6 | 96.3 | 97.0 | 96.8 |
| Drinking Milk Products | 96.4 | 97.2 | 97.4 | 97.1 | 96.9 |
| Yoghurt and Sour Milk Products | 95.6 | 94.9 | 94.9 | 95.2 | 96.1 |
| Other Dairy | 97.8 | 97.8 | 97.7 | 97.9 | 97.8 |
| **Snacks** |  |  |  |  |  |
| Chocolate Confectionery | 83.5 | 85.8 | 86.9 | 83.9 | 84.6 |
| Gum | 99.8 | 99.8 | 99.8 | 99.7 | 99.7 |
| Sugar Confectionery | 93.1 | 93.1 | 92.9 | 94.5 | 94.0 |
| Savoury Snacks | 95.0 | 94.1 | 92.1 | 91.9 | 92.3 |
| Sweet Biscuits, Snack Bars and Fruit Snacks | 93.2 | 93.2 | 91.3 | 88.7 | 89.8 |
| **Staple Foods** |  |  |  |  |  |
| Baked Goods | 94.1 | 94.1 | 94.1 | 94.8 | 95.9 |
| Breakfast Cereals | 95.3 | 93.7 | 94.6 | 94.1 | 94.8 |
| Processed Fruit and Vegetables | 95.0 | 96.2 | 95.7 | 96.9 | 96.5 |
| Processed Meat, Seafood and Alternatives to Meat | 94.1 | 94.4 | 94.7 | 95.6 | 95.8 |
| Rice, Pasta and Noodles | 93.3 | 95.0 | 95.1 | 96.7 | 97.4 |
| **Soft Drinks** |  |  |  |  |  |
| Bottled Water | 98.4 | 98.6 | 99.2 | 99.0 | 99.1 |
| Carbonates | 95.6 | 96.5 | 96.5 | 96.4 | 96.7 |
| Concentrates | 98.5 | 99.2 | 99.7 | 99.7 | 99.9 |
| Juice | 97.2 | 96.5 | 96.3 | 95.7 | 94.3 |
| RTD Coffee | 100.0 | 100.0 | 98.8 | 99.2 | 99.3 |
| RTD Tea | 100.0 | 100.0 | 100.0 | 99.1 | 99.3 |
| Energy Drinks | 98.8 | 99.2 | 99.3 | 98.7 | 99.3 |
| Sports Drinks | 99.6 | 99.7 | 100.0 | 100.0 | 100.0 |
| **Total** | **95.0** | **95.4** | **95.3** | **95.4** | **95.8** |

**Figure S1.** Front-of-package warning labels used in the Chilean Law of Food Labelling and Advertising.


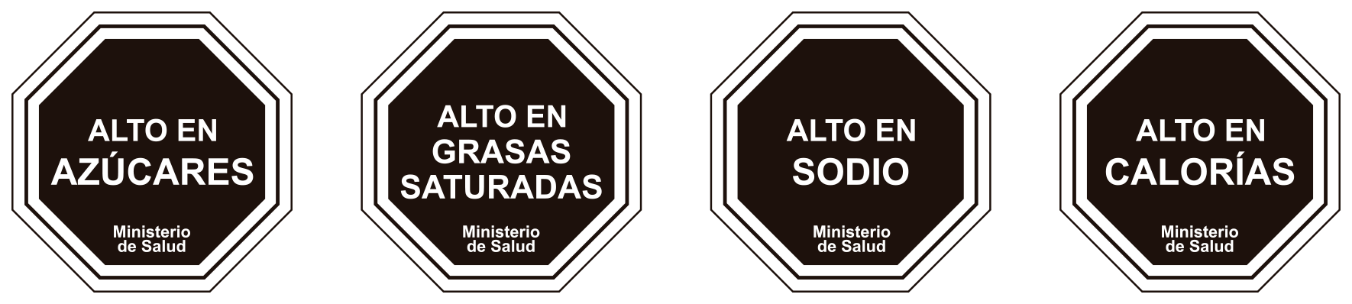


From left to right: (1) high in sugars; (2) high in saturated fats; (3) high in sodium; and (4) high in calories. All labels exhibit “Ministry of Health” at the b the octagon.

**Figure S2.** Flow chart describing products excluded from the analytical sample for sensitivity analyses.

**
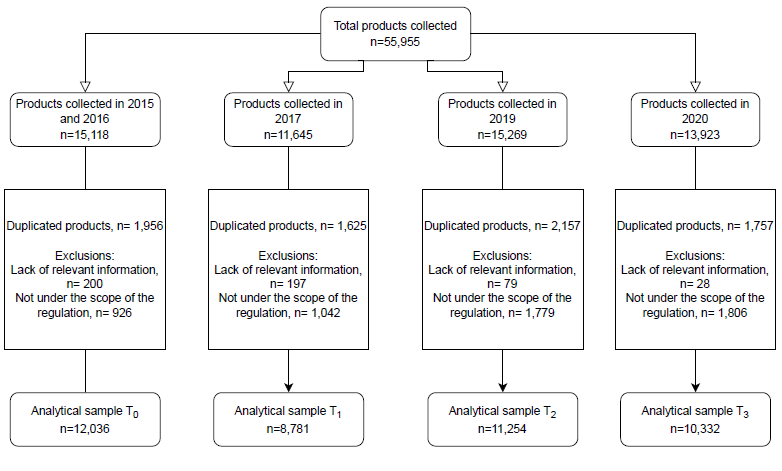
**

Legend: T0: preimplementation period; T1: postimplementation of the 1st phase of the law; T2: postimplementation of the 2nd phase of the law; T3: postimplementation of the 3rd phase of the law.

**Table S2**. Changes in the proportion of "high on" energy and nutrients of concern (or any high in) before (T0) and after each phase of Chile's Law (T1, T2, T3) for solid and liquid products in the full food supply, repeated cross-sectional analysis.

|  | **2016 (T_0_)** | **2017 (T_1_)** | **2019 (T_2_)** | **2020 (T_3_)** | **Difference (T0 vs T3)** | | **p-trend**** |
| --- | --- | --- | --- | --- | --- | --- | --- |
|  |  |  |  |  | **Absolute** | **p-value*** |  |
| **Overall** | N = 12,036 | N = 8,781 | N = 11,254 | N = 10,332 |  |  |  |
| Any "High in"^abcdef^ | 8,702 (72.3%) | 5,996 (68.3%) | 7,072 (62.8%) | 6,274 (60.7%) | -11.6 | <0.001 | <0.001 |
| High in Energy^abcde^ | 5,055 (42.0%) | 3,902 (44.4%) | 4,310 (38.3%) | 3,982 (38.5%) | -3.5 | <0.001 | <0.001 |
| High in Sugars^abcdef^ | 4,742 (39.4%) | 3,623 (41.3%) | 3,974 (35.3%) | 3,439 (33.3%) | -6.1 | <0.001 | <0.001 |
| High in Saturated Fats^bcde^ | 3,477 (28.9%) | 2,602 (29.6%) | 2,993 (26.6%) | 2,678 (25.9%) | -3.0 | <0.001 | <0.001 |
| High in Sodium^abcdf^ | 3,359 (27.9%) | 1,956 (22.3%) | 2,662 (23.7%) | 2,204 (21.3%) | -6.6 | <0.001 | <0.001 |
| **Solids** | N = 9,129 | N = 6,854 | N = 8,808 | N = 8,124 |  |  |  |
| Any "High in"^abcdef^ | 6,965 (76.3%) | 5,061 (73.8%) | 6,258 (71.0%) | 5,525 (68.0%) | -8.3 | <0.001 | <0.001 |
| High in Energy^abcde^ | 4,317 (47.3%) | 3,398 (49.6%) | 3,934 (44.7%) | 3,683 (45.3%) | -2.0 | 0.010 | <0.001 |
| High in Sugars^acdef^ | 3,460 (37.9%) | 2,911 (42.5%) | 3,403 (38.6%) | 2,956 (36.4%) | -1.5 | 0.040 | <0.001 |
| High in Saturated Fats^bcde^ | 3,113 (34.1%) | 2,341 (34.2%) | 2,841 (32.3%) | 2,543 (31.3%) | -2.8 | <0.001 | <0.001 |
| High in Sodium^abcdf^ | 2,889 (31.6%) | 1,715 (25.0%) | 2,390 (27.1%) | 1,930 (23.8%) | -7.8 | <0.001 | <0.001 |
| **Liquids** | N = 2,907 | N = 1,927 | N = 2,446 | N = 2,208 |  |  |  |
| Any "High in"^abcde^ | 1,737 (59.8%) | 935 (48.5%) | 814 (33.3%) | 749 (33.9%) | -25.9 | <0.001 | <0.001 |
| High in Energy^bcde^ | 738 (25.4%) | 504 (26.2%) | 376 (15.4%) | 299 (13.5%) | -11.9 | <0.001 | <0.001 |
| High in Sugars^abcde^ | 1,282 (44.1%) | 712 (36.9%) | 571 (23.3%) | 483 (21.9%) | -22.2 | <0.001 | <0.001 |
| High in Saturated Fats^bcde^ | 364 (12.5%) | 261 (13.5%) | 152 (6.2%) | 135 (6.1%) | -6.4 | <0.001 | <0.001 |
| High in Sodium^abc^ | 470 (16.2%) | 241 (12.5%) | 272 (11.1%) | 274 (12.4%) | -3.8 | <0.001 | <0.001 |
| **Only beverages and milks & milk-based drinks** | N = 1,788 | N = 1,193 | N = 1,714 | N = 1,543 |  |  |  |
| Any "High in"^abcde^ | 684 (38.3%) | 280 (23.5%) | 217 (12.7%) | 207 (13.4%) | -24.9 | <0.001 | <0.001 |
| High in Energy^bcde^ | 48 (2.7%) | 35 (2.9%) | 11 (0.6%) | 7 (0.5%) | -2.2 | <0.001 | <0.001 |
| High in Sugars^abcde^ | 663 (37.1%) | 272 (22.8%) | 207 (12.1%) | 199 (12.9%) | -24.2 | <0.001 | <0.001 |
| High in Saturated Fats^ad^ | 1 (0.1%) | 6 (0.5%) | 1 (0.1%) | 1 (0.1%) | 0.0 | 0.899 | 0.119 |
| High in Sodium | 17 (1.0%) | 8 (0.7%) | 9 (0.5%) | 7 (0.5%) | -0.5 | 0.107 | 0.127 |
| **Other liquids** | N = 1,119 | N = 734 | N = 732 | N = 665 |  |  |  |
| Any "High in"^abcde^ | 1,053 (94.1%) | 655 (89.2%) | 597 (81.6%) | 542 (81.5%) | -12.6 | <0.001 | <0.001 |
| High in Energy^bcdef^ | 690 (61.7%) | 469 (63.9%) | 365 (49.9%) | 292 (43.9%) | -17.8 | <0.001 | <0.001 |
| High in Sugars^abcdef^ | 619 (55.3%) | 440 (59.9%) | 364 (49.7%) | 284 (42.7%) | -12.6 | <0.001 | <0.001 |
| High in Saturated Fats^bcde^ | 363 (32.4%) | 255 (34.7%) | 151 (20.6%) | 134 (20.2%) | -12.2 | <0.001 | <0.001 |
| High in Sodium^abe^ | 453 (40.5%) | 233 (31.7%) | 263 (35.9%) | 267 (40.2%) | -0.3 | 0.892 | 0.192 |

Values represent the sample size and the proportion of regulated products.

Cutoffs correspond to the limits on the amount of energy or nutrient of concern for the full implementation of the law (i.e., for solids, per 100g: 275 kcal of energy, 10 g of sugars, 4 g of saturated fats, 400 mg of sodium; for liquids, per 100 mL: 70 kcal of energy, 5 g of sugars, 3 g of saturated fats, 100 mg of sodium).

T0: preimplementation period, January to February 2015 + January to February 2016 (n = 12,036); T1: postimplementation of the 1st phase of the law, January to February 2017 (n = 8,781); T2: postimplementation of the 2nd phase of the law, January to February 2019 (n = 11,254); T3: postimplementation of the 3rd phase of the law, January to February 2020 (n = 10,332).

a = P-value < 0.05. Comparisons between T0 and T1 were made by contrasting estimated marginal means (EMMs) from Firth's bias-reduced logistic regression.
b = P-value < 0.05. Comparisons between T0 and T2 were made by contrasting estimated marginal means (EMMs) from Firth's bias-reduced logistic regression.
c = P-value < 0.05. Comparisons between T0 and T3 were made by contrasting estimated marginal means (EMMs) from Firth's bias-reduced logistic regression.
d = P-value < 0.05. Comparisons between T1 and T2 were made by contrasting estimated marginal means (EMMs) from Firth's bias-reduced logistic regression.
e = P-value < 0.05. Comparisons between T1 and T3 were made by contrasting estimated marginal means (EMMs) from Firth's bias-reduced logistic regression.
f = P-value < 0.05. Comparisons between T2 and T3 were made by contrasting estimated marginal means (EMMs) from Firth's bias-reduced logistic regression.

* P-value < 0.05. Comparisons between T0 and T3 were made by contrasting estimated marginal means (EMMs) from Firth's bias-reduced logistic regression.

** P-value for Cochrane Armitage Test for trend.

**Table S3.** Changes in the proportion of "high on" energy and nutrients of concern (or any high in) before (T0) and after each phase of Chile's Law (T1, T2, T3) by food group in the full food supply, repeated cross-sectional analysis.

|  | **2016 (T_0_)** | **2017 (T_1_)** | **2019 (T_2_)** | **2020 (T_3_)** | **Difference (T0 vs T3)** | | **p-trend**** |
| --- | --- | --- | --- | --- | --- | --- | --- |
|  |  |  |  |  | **Absolute** | **p-value*** |  |
| **Beverages** | N = 1,579 | N = 1,302 | N = 1,706 | N = 1,433 |  |  |  |
| Any "High in"^abcde^ | 506 (32.0%) | 282 (21.7%) | 213 (12.5%) | 200 (14.0%) | -18 | <0.001 | <0.001 |
| High in Energy^acdef^ | 19 (1.2%) | 35 (2.7%) | 13 (0.8%) | 3 (0.2%) | -1.0 | 0.005 | <0.001 |
| High in Sugars^abcde^ | 484 (30.7%) | 275 (21.1%) | 200 (11.7%) | 192 (13.4%) | -17.3 | <0.001 | <0.001 |
| High in Saturated Fats^a^ | 1 (0.1%) | 7 (0.5%) | 3 (0.2%) | 0 (0.0%) | -0.1 | 0.540 | 0.236 |
| High in Sodium | 16 (1.0%) | 8 (0.6%) | 8 (0.5%) | 7 (0.5%) | -0.5 | 0.115 | 0.057 |
| **Milks & milk-based drinks** | N = 344 | N = 163 | N = 282 | N = 240 |  |  |  |
| Any "High in"^abc^ | 186 (54.1%) | 15 (9.2%) | 16 (5.7%) | 12 (5.0%) | -49.1 | <0.001 | <0.001 |
| High in Energy^abc^ | 32 (9.3%) | 3 (1.8%) | 5 (1.8%) | 4 (1.7%) | -7.6 | <0.001 | <0.001 |
| High in Sugars^abc^ | 184 (53.5%) | 14 (8.6%) | 15 (5.3%) | 12 (5.0%) | -48.5 | <0.001 | <0.001 |
| High in Saturated Fats | 1 (0.3%) | 0 (0.0%) | 1 (0.4%) | 1 (0.4%) | 0.1 | 0.756 | 0.657 |
| High in Sodium | 3 (0.9%) | 0 (0.0%) | 1 (0.4%) | 0 (0.0%) | -0.9 | 0.292 | 0.156 |
| **Yogurts** | N = 488 | N = 417 | N = 369 | N = 312 |  |  |  |
| Any "High in"^abcef^ | 208 (42.6%) | 147 (35.3%) | 123 (33.3%) | 21 (6.7%) | -35.9 | <0.001 | <0.001 |
| High in Energy | 7 (1.4%) | 1 (0.2%) | 7 (1.9%) | 3 (1.0%) | -0.4 | 0.623 | 0.700 |
| High in Sugars^bcef^ | 196 (40.2%) | 146 (35.0%) | 118 (32.0%) | 17 (5.4%) | -34.8 | <0.001 | <0.001 |
| High in Saturated Fats | 6 (1.2%) | 4 (1.0%) | 8 (2.2%) | 2 (0.6%) | -0.6 | 0.491 | 0.881 |
| High in Sodium | 7 (1.4%) | 0 (0.0%) | 4 (1.1%) | 4 (1.3%) | -0.1 | 0.913 | 0.768 |
| **Breakfast cereals** | N = 447 | N = 317 | N = 383 | N = 421 |  |  |  |
| Any "High in"^bcdef^ | 400 (89.5%) | 286 (90.2%) | 281 (73.4%) | 275 (65.3%) | -24.2 | <0.001 | <0.001 |
| High in Energy^bcdef^ | 395 (88.4%) | 283 (89.3%) | 276 (72.1%) | 268 (63.7%) | -24.7 | <0.001 | <0.001 |
| High in Sugars^bcdef^ | 329 (73.6%) | 221 (69.7%) | 198 (51.7%) | 166 (39.4%) | -34.2 | <0.001 | <0.001 |
| High in Saturated Fats^cde^ | 102 (22.8%) | 83 (26.2%) | 73 (19.1%) | 62 (14.7%) | -8.1 | 0.003 | <0.001 |
| High in Sodium^c^ | 57 (12.8%) | 31 (9.8%) | 37 (9.7%) | 29 (6.9%) | -5.9 | 0.005 | 0.008 |
| **Sweet baked products** | N = 1,210 | N = 1,144 | N = 1,188 | N = 1,222 |  |  |  |
| Any "High in"^acd^ | 1,194 (98.7%) | 1,113 (97.3%) | 1,171 (98.6%) | 1,192 (97.5%) | -1.2 | 0.046 | 0.407 |
| High in Energy^abcf^ | 1,170 (96.7%) | 1,039 (90.8%) | 1,086 (91.4%) | 1,084 (88.7%) | -8 | <0.001 | <0.001 |
| High in Sugars^bf^ | 1,017 (84.0%) | 994 (86.9%) | 1,042 (87.7%) | 1,030 (84.3%) | 0.3 | 0.872 | 0.683 |
| High in Saturated Fats^bce^ | 879 (72.6%) | 792 (69.2%) | 778 (65.5%) | 757 (61.9%) | -10.7 | <0.001 | <0.001 |
| High in Sodium | 70 (5.8%) | 61 (5.3%) | 60 (5.1%) | 51 (4.2%) | -1.6 | 0.070 | 0.078 |
| **Desserts, ice-creams & processed fruits** | N = 1,006 | N = 717 | N = 738 | N = 655 |  |  |  |
| Any "High in"^bcdef^ | 829 (82.4%) | 612 (85.4%) | 572 (77.5%) | 472 (72.1%) | -10.3 | <0.001 | <0.001 |
| High in Energy^bcde^ | 544 (54.1%) | 413 (57.6%) | 297 (40.2%) | 255 (38.9%) | -15.2 | <0.001 | <0.001 |
| High in Sugars^cdef^ | 780 (77.5%) | 576 (80.3%) | 543 (73.6%) | 434 (66.3%) | -11.2 | <0.001 | <0.001 |
| High in Saturated Fats^ade^ | 309 (30.7%) | 288 (40.2%) | 248 (33.6%) | 223 (34.0%) | 3.3 | 0.155 | 0.613 |
| High in Sodium^abcde^ | 21 (2.1%) | 35 (4.9%) | 5 (0.7%) | 4 (0.6%) | -1.5 | 0.027 | <0.001 |
| **Candies & sweet confectionery** | N = 1,493 | N = 1,346 | N = 1,530 | N = 1,422 |  |  |  |
| Any "High in" | 1,263 (84.6%) | 1,108 (82.3%) | 1,283 (83.9%) | 1,173 (82.5%) | -2.1 | 0.126 | 0.373 |
| High in Energy | 1,187 (79.5% | 1,070 (79.5% | 1,240 (81.0% | 1,161 (81.6% | 2.1 | 0.145 | 0.078 |
| High in Sugars^d^ | 1,138 (76.2%) | 1,011 (75.1%) | 1,207 (78.9%) | 1,082 (76.1%) | -0.1 | 0.933 | 0.310 |
| High in Saturated Fats | 643 (43.1%) | 604 (44.9%) | 688 (45.0%) | 622 (43.7%) | 0.6 | 0.714 | 0.695 |
| High in Sodium | 13 (0.9%) | 9 (0.7%) | 13 (0.8%) | 8 (0.6%) | -0.3 | 0.343 | 0.542 |
| **Sweet spreads** | N = 627 | N = 407 | N = 608 | N = 502 |  |  |  |
| Any "High in"^bc^ | 502 (80.1%) | 312 (76.7%) | 448 (73.7%) | 362 (72.1%) | -8 | 0.002 | 0.001 |
| High in Energy^aef^ | 219 (34.9%) | 168 (41.3%) | 240 (39.5%) | 158 (31.5%) | -3.4 | 0.223 | 0.371 |
| High in Sugars^ac^ | 431 (68.7%) | 240 (59.0%) | 391 (64.3%) | 304 (60.6%) | -8.1 | 0.004 | 0.045 |
| High in Saturated Fats | 149 (23.8%) | 94 (23.1%) | 173 (28.5%) | 139 (27.7%) | 3.9 | 0.133 | 0.028 |
| High in Sodium | 12 (1.9%) | 15 (3.7%) | 15 (2.5%) | 11 (2.2%) | 0.3 | 0.732 | 0.946 |
| **Savory baked products** | N = 491 | N = 407 | N = 391 | N = 445 |  |  |  |
| Any "High in"^bcdef^ | 386 (78.6%) | 304 (74.7%) | 247 (63.2%) | 214 (48.1%) | -30.5 | <0.001 | <0.001 |
| High in Energy^bcdef^ | 308 (62.7%) | 255 (62.7%) | 201 (51.4%) | 177 (39.8%) | -22.9 | <0.001 | <0.001 |
| High in Sugars^cde^ | 13 (2.6%) | 15 (3.7%) | 4 (1.0%) | 3 (0.7%) | -1.9 | 0.035 | 0.002 |
| High in Saturated Fats^cde^ | 89 (18.1%) | 80 (19.7%) | 54 (13.8%) | 45 (10.1%) | -8.0 | <0.001 | <0.001 |
| High in Sodium^bcdef^ | 306 (62.3%) | 230 (56.5%) | 172 (44.0%) | 142 (31.9%) | -30.4 | <0.001 | <0.001 |
| **Nuts & snacks** | N = 294 | N = 251 | N = 327 | N = 331 |  |  |  |
| Any "High in"^d^ | 266 (90.5%) | 238 (94.8%) | 286 (87.5%) | 304 (91.8%) | 1.3 | 0.546 | 0.434 |
| High in Energy^ad^ | 248 (84.4%) | 230 (91.6%) | 278 (85.0%) | 296 (89.4%) | 5 | 0.062 | 0.484 |
| High in Sugars | 8 (2.7%) | 4 (1.6%) | 6 (1.8%) | 11 (3.3%) | 0.6 | 0.679 | 0.605 |
| High in Saturated Fats^bcde^ | 121 (41.2%) | 98 (39.0%) | 99 (30.3%) | 96 (29.0%) | -12.2 | 0.002 | <0.001 |
| High in Sodium^aef^ | 160 (54.4%) | 165 (65.7%) | 189 (57.8%) | 164 (49.5%) | -4.9 | 0.225 | 0.055 |
| **Savory spreads, seasonings & dressings** | N = 858 | N = 576 | N = 981 | N = 887 |  |  |  |
| Any "High in"^abc^ | 746 (86.9%) | 456 (79.2%) | 785 (80.0%) | 681 (76.8%) | -10.1 | <0.001 | <0.001 |
| High in Energy^abcd^ | 346 (40.3%) | 140 (24.3%) | 291 (29.7%) | 247 (27.8%) | -12.5 | <0.001 | <0.001 |
| High in Sugars^b^ | 147 (17.1%) | 112 (19.4%) | 223 (22.7%) | 172 (19.4%) | 2.3 | 0.224 | 0.059 |
| High in Saturated Fats^abc^ | 255 (29.7%) | 92 (16.0%) | 185 (18.9%) | 141 (15.9%) | -13.8 | <0.001 | <0.001 |
| High in Sodium^abcf^ | 652 (76.0%) | 405 (70.3%) | 694 (70.7%) | 586 (66.1%) | -9.9 | <0.001 | <0.001 |
| **Cheeses** | N = 559 | N = 450 | N = 508 | N = 428 |  |  |  |
| Any "High in"^bcde^ | 436 (78.0%) | 342 (76.0%) | 349 (68.7%) | 281 (65.7%) | -12.3 | <0.001 | <0.001 |
| High in Energy | 90 (16.1%) | 70 (15.6%) | 68 (13.4%) | 63 (14.7%) | -1.4 | 0.559 | 0.307 |
| High in Sugars | 1 (0.2%) | 0 (0.0%) | 4 (0.8%) | 1 (0.2%) | 0.0 | 0.817 | 0.232 |
| High in Saturated Fats^abc^ | 162 (29.0%) | 95 (21.1%) | 90 (17.7%) | 93 (21.7%) | -7.3 | 0.010 | 0.001 |
| High in Sodium^cde^ | 381 (68.2%) | 316 (70.2%) | 325 (64.0%) | 253 (59.1%) | -9.1 | 0.003 | 0.001 |
| **Ready-to-eat meals** | N = 852 | N = 571 | N = 981 | N = 889 |  |  |  |
| Any "High in" | 431 (50.6%) | 300 (52.5%) | 502 (51.2%) | 441 (49.6%) | -1 | 0.683 | 0.601 |
| High in Energy^bcde^ | 141 (16.5%) | 82 (14.4%) | 97 (9.9%) | 88 (9.9%) | -6.6 | <0.001 | <0.001 |
| High in Sugars | 11 (1.3%) | 14 (2.5%) | 22 (2.2%) | 14 (1.6%) | 0.3 | 0.629 | 0.685 |
| High in Saturated Fats^de^ | 178 (20.9%) | 134 (23.5%) | 185 (18.9%) | 170 (19.1%) | -1.8 | 0.357 | 0.088 |
| High in Sodium | 383 (45.0%) | 242 (42.4%) | 437 (44.5%) | 376 (42.3%) | -2.7 | 0.264 | 0.505 |
| **Sausages** | N = 659 | N = 223 | N = 480 | N = 370 |  |  |  |
| Any "High in"^cef^ | 648 (98.3%) | 221 (99.1%) | 474 (98.8%) | 348 (94.1%) | -4.2 | <0.001 | 0.001 |
| High in Energy | 296 (44.9%) | 88 (39.5%) | 189 (39.4%) | 155 (41.9%) | -3 | 0.350 | 0.176 |
| High in Sugars | 0 (0.0%) | 1 (0.4%) | 0 (0.0%) | 0 (0.0%) | 0.0 | 0.773 | 0.623 |
| High in Saturated Fats | 395 (59.9%) | 149 (66.8%) | 299 (62.3%) | 236 (63.8%) | 3.9 | 0.227 | 0.339 |
| High in Sodium^abc^ | 642 (97.4%) | 208 (93.3%) | 443 (92.3%) | 340 (91.9%) | -5.5 | <0.001 | <0.001 |
| **Non-sausages meat products** | N = 921 | N = 384 | N = 692 | N = 669 |  |  |  |
| Any "High in"^abcde^ | 495 (53.7%) | 165 (43.0%) | 247 (35.7%) | 208 (31.1%) | -22.6 | <0.001 | <0.001 |
| High in Energy^bc^ | 49 (5.3%) | 18 (4.7%) | 22 (3.2%) | 20 (3.0%) | -2.3 | 0.029 | 0.007 |
| High in Sugars | 2 (0.2%) | 0 (0.0%) | 1 (0.1%) | 1 (0.1%) | -0.1 | 0.853 | 0.832 |
| High in Saturated Fats^bcde^ | 187 (20.3%) | 79 (20.6%) | 109 (15.8%) | 91 (13.6%) | -6.7 | <0.001 | <0.001 |
| High in Sodium^abcdef^ | 430 (46.7%) | 136 (35.4%) | 184 (26.6%) | 139 (20.8%) | -25.9 | <0.001 | <0.001 |
| **Soups** | N = 208 | N = 106 | N = 90 | N = 106 |  |  |  |
| Any "High in"^abc^ | 206 (99.0%) | 95 (89.6%) | 75 (83.3%) | 90 (84.9%) | -14.1 | <0.001 | <0.001 |
| High in Energy^a^ | 4 (1.9%) | 7 (6.6%) | 0 (0.0%) | 0 (0.0%) | -1.9 | 0.302 | 0.055 |
| High in Sugars | 1 (0.5%) | 0 (0.0%) | 0 (0.0%) | 0 (0.0%) | -0.5 | 0.792 | 0.336 |
| High in Saturated Fats | 0 (0.0%) | 3 (2.8%) | 0 (0.0%) | 0 (0.0%) | 0 | 0.738 | 0.545 |
| High in Sodium^abc^ | 206 (99.0%) | 95 (89.6%) | 75 (83.3%) | 90 (84.9%) | -14.1 | <0.001 | <0.001 |

Values represent the sample size and the proportion of regulated products.

Cutoffs correspond to the limits on the amount of energy or nutrient of concern for the full implementation of the law (i.e., for solids, per 100g: 275 kcal of energy, 10 g of sugars, 4 g of saturated fats, 400 mg of sodium; for liquids, per 100 mL: 70 kcal of energy, 5 g of sugars, 3 g of saturated fats, 100 mg of sodium).

T0: preimplementation period, January to February 2015 + January to February 2016 (n = 12,036); T1: postimplementation of the 1st phase of the law, January to February 2017 (n = 8,781); T2: postimplementation of the 2nd phase of the law, January to February 2019 (n = 11,254); T3: postimplementation of the 3rd phase of the law, January to February 2020 (n = 10,332).

a = P-value < 0.05. Comparisons between T0 and T1 were made by contrasting estimated marginal means (EMMs) from Firth's bias-reduced logistic regression.
b = P-value < 0.05. Comparisons between T0 and T2 were made by contrasting estimated marginal means (EMMs) from Firth's bias-reduced logistic regression.
c = P-value < 0.05. Comparisons between T0 and T3 were made by contrasting estimated marginal means (EMMs) from Firth's bias-reduced logistic regression.
d = P-value < 0.05. Comparisons between T1 and T2 were made by contrasting estimated marginal means (EMMs) from Firth's bias-reduced logistic regression.
e = P-value < 0.05. Comparisons between T1 and T3 were made by contrasting estimated marginal means (EMMs) from Firth's bias-reduced logistic regression.
f = P-value < 0.05. Comparisons between T2 and T3 were made by contrasting estimated marginal means (EMMs) from Firth's bias-reduced logistic regression.

* P-value < 0.05. Comparisons between T0 and T3 were made by contrasting estimated marginal means (EMMs) from Firth's bias-reduced logistic regression.

** P-value for Cochrane Armitage Test for trend.

**Table S4.** Changes in quartiles of energy and nutrients of concern before (T0) and after each phase of Chile's Law (T1, T2, T3) for solid and liquid and by food and beverage group in the full food supply, repeated cross-sectional analysis.

|  | **ENERGY** | | | | **TOTAL SUGARS** | | | | **SATURATED FATS** | | | | **SODIUM** | | | |
| --- | --- | --- | --- | --- | --- | --- | --- | --- | --- | --- | --- | --- | --- | --- | --- | --- |
|  | **(kcal/100g-mL)** | | | | **(g/100g-mL)** | | | | **(g/100g-mL)** | | | | **(mg/100g-mL)** | | | |
|  | **2016 (T_0_)** | **2017 (T_1_)** | **2019 (T_2_)** | **2020 (T_3_)** | **2016 (T_0_)** | **2017 (T_1_)** | **2019 (T_2_)** | **2020 (T_3_)** | **2016 (T_0_)** | **2017 (T_1_)** | **2019 (T_2_)** | **2020 (T_3_)** | **2016 (T_0_)** | **2017 (T_1_)** | **2019 (T_2_)** | **2020 (T_3_)** |
| **Overall** | | | | | | | | | | | | | | | | |
| p25 | 60.0 | 59.0 | 57.0 | 59.0 | 0.7 | **0.9** | **0.5** | **0.5** | 0.0 | 0.0 | 0.0 | 0.0 | 30.8 | 30.0 | **25.0** | 30.0 |
| p50 | 205.0 | **236.0** | 213.0 | **221.0** | 5.4 | **6.0** | **4.7** | **4.6** | 1.4 | 1.6 | 1.5 | 1.5 | 133.0 | **100.0** | **113.0** | 127.0 |
| p75 | 387.0 | **398.0** | 384.0 | 384.0 | 22.0 | **25.1** | 22.0 | 23.0 | 7.5 | 7.5 | **6.5** | **6.5** | 427.0 | **362.0** | **392.0** | **384.0** |
| **Solids** | | | | | | | | | | | | | | | | |
| p25 | 117.0 | 122.8 | **125.0** | **137.0** | 0.6 | **1.0** | 0.5 | 0.7 | 0.0 | 0.0 | 0.0 | 0.0 | 55.0 | **48.0** | **49.0** | 55.0 |
| p50 | 292.0 | **314.0** | 287.0 | 288.9 | 5.6 | **7.7** | 5.5 | 5.4 | 2.7 | 2.8 | 2.8 | 2.7 | 228.0 | **163.0** | **200.0** | **210.0** |
| p75 | 423.2 | **434.0** | **413.0** | **413.0** | 29.0 | **32.0** | 29.5 | 30.0 | 9.6 | 9.7 | **9.0** | **9.0** | 504.0 | **414.0** | **448.0** | **400.0** |
| **Liquids** | | | | | | | | | | | | | | | | |
| p25 | 16.7 | **12.9** | **9.2** | **8.0** | 1.4 | **0.5** | **0.5** | **0.3** | 0.0 | 0.0 | 0.0 | 0.0 | 9.0 | **7.0** | **7.0** | **6.0** |
| p50 | 39.0 | 38.0 | **27.6** | **25.6** | 5.1 | 4.8 | **4.3** | **4.2** | 0.0 | 0.0 | 0.0 | 0.0 | 30.0 | 29.0 | **22.0** | **22.0** |
| p75 | 77.0 | 85.0 | **52.0** | **50.0** | 11.0 | 11.5 | **7.6** | **6.9** | 0.9 | 0.7 | **0.1** | **0.1** | 63.0 | 60.0 | **55.0** | **55.0** |
| **Only beverages and milks & milk-based drinks** | | | | | | | | | | | | | | | | |
| p25 | 2.8 | 2.2 | 2.3 | 2.0 | 0.1 | 0.1 | 0.1 | **0.0** | 0.0 | 0.0 | 0.0 | 0.0 | 3.7 | **1.9** | **2.3** | **1.9** |
| p50 | 18.5 | 21.0 | 19.0 | 17.5 | 3.6 | 3.0 | **2.9** | **2.8** | 0.0 | 0.0 | 0.0 | 0.0 | 9.0 | 9.0 | **10.0** | **10.0** |
| p75 | 41.1 | 42.0 | **34.0** | **32.5** | 8.7 | 7.7 | **5.0** | **5.0** | 0.0 | 0.0 | 0.0 | 0.0 | 17.5 | 20.0 | **21.0** | **21.0** |
| **Other liquids** | | | | | | | | | | | | | | | | |
| p25 | 37.0 | 36.0 | **32.0** | **29.0** | 2.7 | 2.5 | 2.1 | **1.4** | 0.0 | 0.0 | 0.0 | 0.0 | 40.1 | 40.0 | 40.0 | 40.0 |
| p50 | 73.0 | 84.8 | **52.0** | **50.0** | 6.7 | 6.2 | **4.8** | **4.7** | 0.9 | 1.0 | **0.4** | **0.2** | 60.0 | 56.0 | **56.0** | 59.0 |
| p75 | 144.0 | 159.0 | **110.0** | **100.0** | 14.8 | **18.0** | **12.2** | **11.5** | 3.7 | 4.0 | **2.0** | **1.9** | 276.2 | **125.7** | 181.9 | 280.0 |
| **Beverages** | | | | | | | | | | | | | | | | |
| p25 | 2.1 | **1.0** | **1.2** | **1.6** | 0.1 | **0.0** | **0.0** | **0.0** | 0.0 | 0.0 | 0.0 | 0.0 | 2.4 | **1.0** | **1.1** | **1.0** |
| p50 | 17.0 | 15.0 | **13.0** | **14.0** | 3.0 | 2.3 | **2.1** | **2.3** | 0.0 | 0.0 | 0.0 | 0.0 | 9.0 | **8.0** | **8.0** | 9.0 |
| p75 | 41.0 | 41.3 | **29.0** | **29.0** | 8.0 | **6.7** | **4.9** | **4.9** | 0.0 | 0.0 | 0.0 | 0.0 | 18.0 | 19.7 | 20.0 | **20.0** |
| **Milks & milk-based drinks** | | | | | | | | | | | | | | | | |
| p25 | 38.0 | 39.0 | **35.7** | 36.0 | 4.8 | **4.4** | **4.4** | **4.4** | 0.1 | **0.0** | **0.0** | **0.0** | 44.0 | **49.0** | 44.7 | 43.0 |
| p50 | 48.0 | 46.0 | 46.0 | 48.0 | 6.0 | **4.7** | **4.8** | **4.7** | 0.7 | **0.4** | 0.7 | 0.8 | 52.1 | 51.0 | 53.0 | 54.0 |
| p75 | 59.0 | **54.0** | **52.0** | **52.0** | 7.3 | **5.0** | **5.0** | **5.0** | 1.0 | 1.0 | 1.0 | 1.0 | 66.0 | **59.0** | 65.0 | 65.0 |
| **Yogurts** | | | | | | | | | | | | | | | | |
| p25 | 53.0 | 51.0 | 54.0 | 54.5 | 5.7 | 5.5 | 5.6 | 5.5 | 0.1 | 0.0 | 0.1 | 0.1 | 46.0 | 47.0 | 44.0 | 44.0 |
| p50 | 80.0 | 70.0 | 81.0 | 75.0 | 8.5 | 8.2 | 8.8 | 8.0 | 0.8 | 0.7 | 1.0 | 1.0 | 55.0 | 55.0 | **52.0** | 55.0 |
| p75 | 97.0 | 97.0 | 99.0 | 93.0 | 13.3 | 13.1 | 12.6 | **9.9** | 1.5 | 1.6 | **2.0** | **1.8** | 65.0 | **60.0** | **61.0** | 65.0 |
| **Breakfast cereals** | | | | | | | | | | | | | | | | |
| p25 | 364.0 | **348.0** | **339.0** | **332.0** | 13.0 | 12.5 | **3.5** | **2.2** | 1.0 | 0.9 | **0.5** | **0.5** | 71.4 | 44.0 | **17.0** | **19.9** |
| p50 | 381.0 | 377.0 | **365.0** | **364.0** | 23.8 | **20.0** | **14.0** | **9.6** | 2.0 | 2.0 | **1.7** | **1.5** | 180.0 | **124.0** | **123.0** | **102.0** |
| p75 | 404.0 | 400.0 | **390.0** | 391.4 | 30.0 | 28.6 | 28.0 | 28.3 | 4.0 | 4.4 | 3.8 | 3.6 | 303.0 | 280.0 | 271.0 | **254.0** |
| **Sweet baked products** | | | | | | | | | | | | | | | | |
| p25 | 396.0 | **365.0** | **364.0** | **349.0** | 23.0 | **21.9** | 21.8 | **21.1** | 4.3 | 3.8 | **3.6** | **3.3** | 130.0 | **114.0** | **119.0** | 120.0 |
| p50 | 450.0 | **434.0** | **424.0** | **418.0** | 29.4 | 29.3 | 29.5 | 29.0 | 8.8 | **8.0** | **7.3** | **7.1** | 200.0 | 188.0 | 193.0 | 198.0 |
| p75 | 498.0 | **487.0** | **483.0** | **480.0** | 36.0 | 36.0 | 36.3 | 36.0 | 12.6 | 12.0 | **11.7** | 12.0 | 290.0 | **273.0** | 278.0 | 288.0 |
| **Desserts, ice-creams & processed fruits** | | | | | | | | | | | | | | | | |
| p25 | 76.0 | **81.0** | 75.0 | 76.0 | 12.0 | **12.8** | 11.4 | **10.3** | 0.0 | 0.0 | 0.0 | 0.0 | 13.5 | **18.0** | 13.6 | 12.0 |
| p50 | 105.0 | **124.0** | **115.0** | **122.0** | 16.0 | **17.4** | 15.0 | 15.8 | 0.6 | **2.1** | 1.3 | 1.3 | 36.0 | **43.0** | 39.0 | 39.2 |
| p75 | 165.0 | **203.0** | **216.0** | **220.0** | 20.6 | **21.9** | **22.0** | **22.2** | 4.1 | **5.0** | **4.8** | **5.0** | 56.0 | **67.0** | **62.0** | **65.0** |
| **Candies & sweet confectionery** | | | | | | | | | | | | | | | | |
| p25 | 323.0 | 332.0 | **343.0** | **340.0** | 25.0 | 25.3 | 28.0 | 28.0 | 0.0 | 0.0 | 0.0 | 0.0 | 19.0 | 16.0 | **12.0** | **13.0** |
| p50 | 414.0 | 424.0 | **436.0** | 416.0 | 50.0 | 50.0 | 49.0 | 50.0 | 3.5 | 4.0 | 3.9 | 3.2 | 49.5 | 48.0 | **42.0** | 46.0 |
| p75 | 530.0 | 528.0 | 531.0 | 527.0 | 60.4 | 61.0 | 60.0 | 62.0 | 17.2 | 17.4 | 18.0 | 16.9 | 104.0 | 100.0 | 99.0 | **95.0** |
| **Sweet spreads** | | | | | | | | | | | | | | | | |
| p25 | 119.0 | 120.0 | 102.9 | 94.0 | 7.7 | 5.2 | 6.2 | 5.4 | 0.0 | 0.0 | 0.0 | 0.0 | 10.9 | 12.0 | 9.6 | 11.0 |
| p50 | 222.0 | 238.0 | **241.0** | 216.0 | 36.3 | **21.0** | **27.7** | **25.3** | 0.0 | 0.0 | 0.0 | 0.0 | 23.0 | 26.7 | 25.0 | 23.0 |
| p75 | 310.0 | 340.0 | **335.0** | 307.0 | 54.0 | 54.0 | 52.7 | **51.0** | 6.1 | 7.6 | 7.4 | 6.3 | 56.1 | **71.0** | **76.0** | **70.0** |
| **Savory baked products** | | | | | | | | | | | | | | | | |
| p25 | 269.0 | 265.0 | **256.0** | **249.0** | 1.3 | **1.8** | 1.7 | 1.6 | 0.6 | 0.5 | 0.6 | 0.5 | 377.0 | 350.0 | **338.0** | **336.0** |
| p50 | 339.0 | 314.0 | **299.0** | **274.0** | 3.0 | 3.1 | 3.0 | 2.8 | 1.3 | 1.4 | 1.4 | 1.2 | 464.0 | 433.0 | **396.0** | **392.0** |
| p75 | 415.8 | 421.0 | **401.0** | **386.3** | 5.0 | 5.0 | 4.8 | **4.5** | 3.2 | 3.3 | 3.0 | 2.8 | 657.0 | 665.0 | **551.0** | **494.0** |
| **Nuts & snacks** | | | | | | | | | | | | | | | | |
| p25 | 482.0 | 480.0 | 490.0 | 481.0 | 0.6 | 1.5 | 1.4 | **1.6** | 3.1 | 3.0 | 2.9 | **2.6** | 262.0 | 337.0 | 308.0 | **329.0** |
| p50 | 518.1 | 513.0 | 513.0 | **505.0** | 3.6 | 3.3 | 3.6 | 3.5 | 4.0 | 4.0 | 3.8 | **3.5** | 455.0 | 492.0 | 457.0 | 403.0 |
| p75 | 560.0 | 548.0 | 548.0 | **532.0** | 7.1 | 6.5 | 6.6 | 6.4 | 6.7 | 5.8 | **5.0** | **5.1** | 607.0 | 652.0 | 580.0 | 651.0 |
| **Savory spreads, seasonings & dressings** | | | | | | | | | | | | | | | | |
| p25 | 50.0 | 47.0 | 52.0 | 46.0 | 0.3 | 0.3 | **0.8** | 0.5 | 0.0 | 0.0 | 0.0 | 0.0 | 386.6 | 359.0 | 367.0 | 354.8 |
| p50 | 125.0 | **96.0** | 116.0 | 107.0 | 2.7 | 3.2 | **3.3** | 3.0 | 0.0 | 0.0 | 0.0 | 0.0 | 590.0 | 590.0 | 607.0 | 605.0 |
| p75 | 362.0 | **211.0** | **260.0** | **244.0** | 6.5 | 7.0 | **8.4** | 7.5 | 10.0 | **3.0** | **3.4** | **3.2** | 1020.0 | 1106.0 | **1176.0** | 1132.0 |
| **Cheeses** | |  | | | | | | | | | | | |  |  |  |
| p25 | 266.0 | **292.0** | **286.0** | 275.0 | 0.0 | 0.0 | 0.0 | 0.0 | 13.3 | **14.9** | **15.0** | 14.3 | 375.0 | 378.0 | 375.0 | 356.0 |
| p50 | 320.0 | **334.0** | **336.0** | 327.0 | 0.5 | 0.5 | 0.5 | 0.5 | 16.6 | 17.3 | **18.0** | 17.1 | 590.0 | 560.0 | **508.0** | **500.0** |
| p75 | 359.0 | 369.0 | 368.0 | 360.0 | 2.7 | **1.7** | **1.4** | **1.5** | 19.3 | 19.9 | 20.0 | 20.0 | 847.0 | **730.0** | **751.0** | 770.0 |
| **Ready-to-eat meals** | | | | | | | | | | | | | | | | |
| p25 | 68.4 | 67.0 | 76.8 | 69.0 | 0.5 | 0.7 | 0.5 | 0.5 | 0.0 | 0.0 | 0.0 | 0.0 | 253.6 | 270.0 | 252.4 | 257.0 |
| p50 | 132.0 | 148.0 | **146.0** | 144.0 | 1.4 | 1.6 | 1.4 | 1.4 | 1.1 | 1.2 | **1.7** | 1.5 | 375.0 | 372.0 | 388.0 | 377.0 |
| p75 | 245.0 | 249.0 | 232.0 | 228.0 | 2.9 | 2.8 | 2.7 | 2.7 | 3.6 | 4.2 | 3.8 | 3.8 | 576.0 | 546.0 | 565.1 | 564.0 |
| **Sausages** | | | | | | | | | | | | | | | | |
| p25 | 192.0 | 161.0 | 170.0 | 172.0 | 0.2 | 0.4 | **0.5** | 0.1 | 4.6 | 4.0 | 3.9 | 3.9 | 780.0 | **713.0** | **605.0** | **669.0** |
| p50 | 266.0 | **244.0** | 258.0 | 262.0 | 0.5 | 0.5 | 0.5 | 0.5 | 8.1 | **6.9** | 7.5 | 8.4 | 929.0 | **776.0** | **783.4** | **784.0** |
| p75 | 336.0 | **310.0** | **319.0** | 329.0 | 1.3 | **1.0** | **0.8** | **0.5** | 11.0 | **10.1** | **10.3** | 11.0 | 1123.0 | **831.3** | **1028.0** | **961.0** |
| **Non-sausages meat products** | | | | | | | | | | | | | | | | |
| p25 | 109.0 | 105.0 | **96.5** | **98.0** | 0.0 | 0.0 | 0.0 | 0.0 | 0.6 | 0.6 | **0.1** | **0.2** | 240.0 | **172.0** | **170.0** | **179.0** |
| p50 | 160.0 | **146.0** | **139.0** | **140.0** | 0.0 | 0.0 | 0.0 | 0.0 | 1.9 | 1.9 | **1.5** | **1.3** | 400.0 | **320.0** | **320.0** | **330.0** |
| p75 | 207.0 | 202.0 | 201.0 | 200.0 | 0.5 | 0.5 | 0.5 | 0.5 | 4.0 | 4.0 | 3.6 | **3.2** | 548.0 | **498.0** | **426.0** | **398.0** |
| **Soups** | | | | | | | | | | | | | | | | |
| p25 | 23.2 | 23.0 | 23.0 | **22.1** | 0.3 | 0.4 | 0.3 | 0.3 | 0.0 | **0.1** | 0.0 | 0.0 | 285.6 | 273.9 | **183.7** | 234.0 |
| p50 | 25.4 | 25.8 | 25.5 | 24.9 | 0.6 | **0.8** | 0.7 | 0.7 | 0.1 | 0.1 | 0.1 | 0.1 | 331.4 | 336.4 | 311.2 | 311.5 |
| p75 | 33.8 | 33.9 | 31.6 | **29.6** | 1.4 | 1.4 | 1.1 | 1.1 | 0.3 | 0.4 | 0.2 | 0.2 | 381.3 | 378.7 | 372.4 | 370.1 |

T0: preimplementation period, January to February 2015 + January to February 2016 (n = 12,036); T1: postimplementation of the 1st phase of the law, January to February 2017 (n = 8,781); T2: postimplementation of the 2nd phase of the law, January to February 2019 (n = 11,254); T3: postimplementation of the 3rd phase of the law, January to February 2020 (n = 10,332).

Quartiles and p-values were obtained from quantile regressions models (one model per nutrient per food or beverage group), using implementation period as independent variable. Significant p-values are bold and represent a p-value <0.05 versus T0.
